# Supplementary material for: Reproducible flaws unveil electrostatic aspects of semiconductor electrochemistry
Source: Nat Commun. 2017 Dec 12;8:2066. doi: 10.1038/s41467-017-02091-1 (PMC5727234; doi:10.1038/s41467-017-02091-1)
Supplement: Supplementary file 1 — Supplementary Information [file 41467_2017_2091_MOESM1_ESM.pdf]

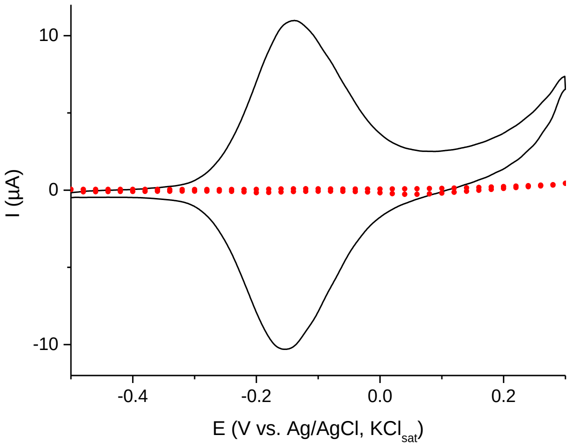

Supplementary Figure 1. **Light-assisted charge-transfer.** Representative cyclic voltammograms (100 mV s^{-1}) for a Si(111) **S-2** sample either under illumination (solid black line) or when shielded from light (red symbols). Electrolyte is aqueous 1.0 M $NaClO_4$.

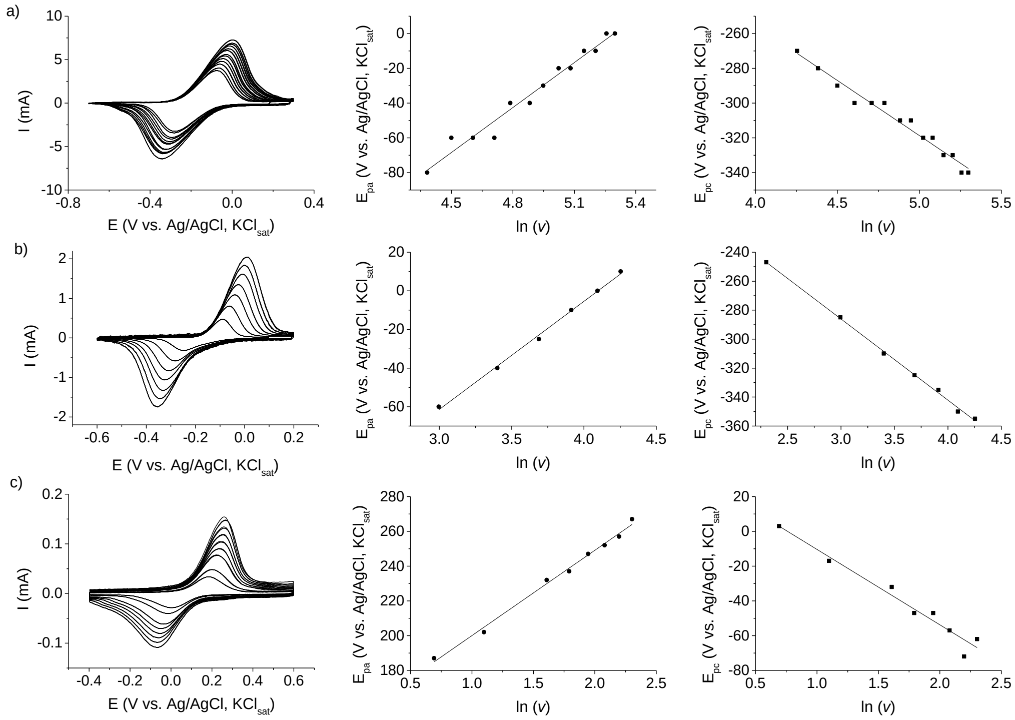

Supplementary Figure 2. **Kinetic study in 1.0 M $HClO_4$ for S-2 samples on the Si(111) lowly-doped n-Si under illumination.** (**(a)** – top row) Kinetic analysis of cyclic voltammograms before anodic oxidation (refined k_{et} = 200 s^{-1}, Model 1), (**(b)** – middle row) after anodic oxidation, when narrow peaks are observed (k_{et} = 80 s^{-1}) and (**(c)** – bottom row) after oxidation when the "narrowing" of the peaks is shielded by slow kinetics (k_{et} = 17 s^{-1}). Plots of $E_{p,a}$ vs ln(v) and $E_{p,c}$ vs. ln(v), with v being the voltage sweep rate, suggest a symmetry coefficient (α) of 0.5. The values of k_{et} for data in rows **(b)** and **(c)** have been obtained from fittings using Models 2 and 3 respectively. In both cases, an estimation of *G* and "s" parameters from cyclic voltammetry has been done using only data at low scan rates, for which the redox kinetics influence is negligible and therefore the nernstian limit can be assumed. The symmetry in the responses of case **(b)** - i.e. narrow but not inverted - for low scan rates (data not shown) suggest that only interactions are required to achieve good fittings between experiment and simulations, and when diode effects are incorporated the shape of the anodic response is different to that observed experimentally. Under these conditions, the following interaction parameters are obtained for voltammograms obtained at a scan rate of 10 V s^{-1}: anodic scan: a = 0.43, s = -0.20, y = -0.25. Cathodic scan: a = 0.43, s = -0.20, y = -0.20. The k_{et} value of obtained for these data is 80 s^{-1}, which is double that obtained by the classical Laviron's analysis of the shift of peak potentials versus the logarithm of the scan rate. If Model 3 is used for data on row **(b)**, good fittings are obtained only if the photocurrent is very high (i.e., $I_L \geq 10^{-3}$ µA), a situation for which $\Theta \rightarrow 0$, and therefore diode effects would not be relevant. The data corresponding to the case in **(c)** are fitted by Model 3. It is apparent that both interactions and diode effects are needed to achieve good fits. With a k_{et} value of 17 s^{-1} and an α of 0.45 the theoretical curves are in good agreement with experimental ones. Moreover, the following parameters are obtained from refining data at a scan rate of 10 V s^{-1}: I_0 = 10^{-5} µA, $I_{L,anodic}$ = 10^{-3} µA, $I_{L,cathodic}$ = 5 × 10^{-4} µA. Anodic scan: a = -0.5, s = 0.20, y = 0.0. Cathodic scan: a = -0.70, s = -0.20, y = -0.60. It should be taken into account that due to the great number of parameters involved, k_{et} values should be considered as estimations only. As indicated above, the classical Laviron's procedure for determining k_{et} based on the shift of the peak potentials with the logarithm of the scan rate allows to obtain k_{et} from the intercept values which are apparently "contaminated" by the influence of both interactions and diode effects. Thus, for data on rows **(b)** and **(c)**, higher k_{et} value are obtained versus those corresponding to only kinetics assumption.

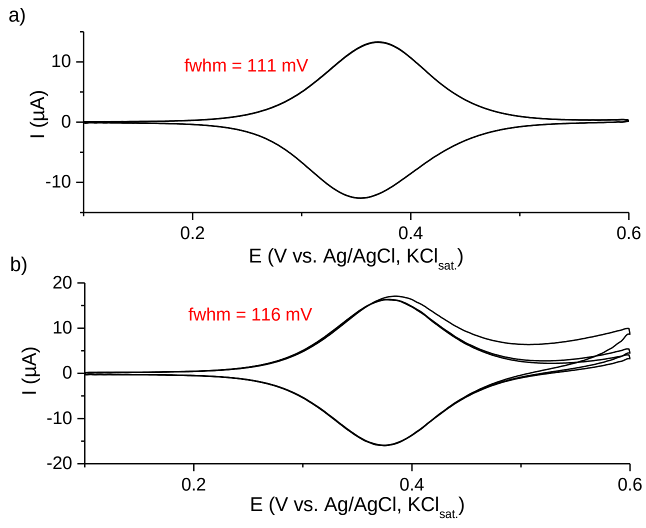

Supplementary Figure 3. **Highly-doped samples**. Cyclic voltammograms (250 mV s^{-1}, 1.0 M $HClO_4$) for as-prepared **S-2** samples on either (a) highly-doped p-Si(111) or (b) highly-doped n-Si(111) substrates. The experimental coverage is $2.5 \cdot \times 10^{-10}$ mol cm^{-2} in both samples and the fwhm's are indicated by labels in figure. Values of *G* inferred from this data are -0.38 **(a)** and -0.47 **(b)**.

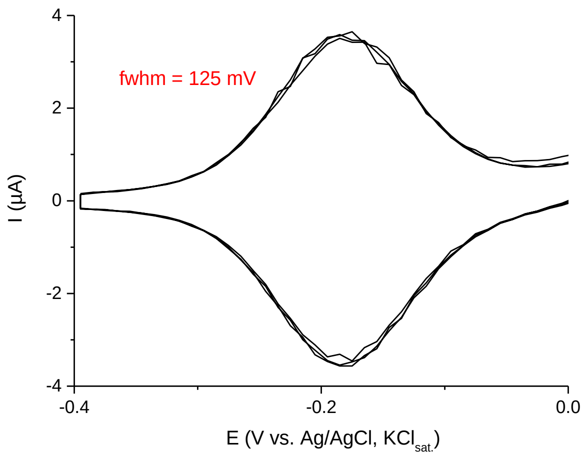

Supplementary Figure 4. **Low-coverage.** Cyclic voltammograms for as-prepared **S-2** samples on Si(111) with low ferrocene coverage. Consecutive cycles, 250 mV s^{-1}, 1.0 M $HClO_4$, $\Gamma = 5.9 \times 10^{-11}$ mol cm^{-2}. The low coverage of ferrocenes is obtained by stopping the click reaction of azide **2** on **S-1** samples after a reaction time of only 1 min. The fwhm value is indicated in figure which corresponds to a *G* value of -0.63.

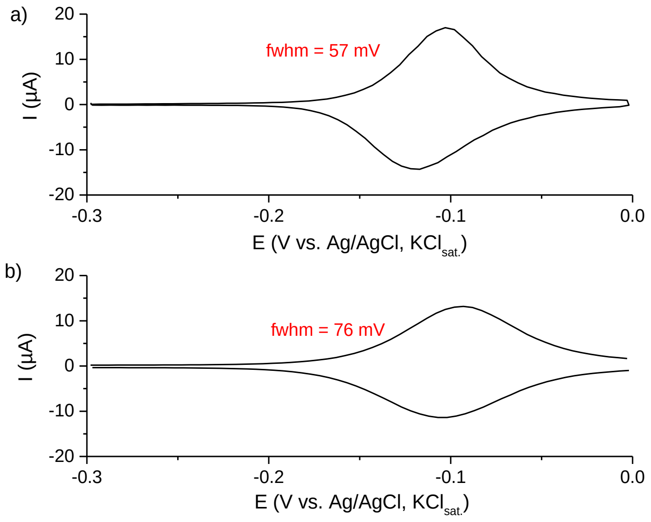

Supplementary Figure 5. **Anodic treatment.** Cyclic voltammograms (250 mV s^{-1}) for **S-2** Si(111) samples after the anodic treatment acquired in (a) 1.0 M $HClO_4$ (pH 0.5) and (b) at in 1.0 M $NaClO_4$ (pH 6.0). Values of fwhm are indicated by labels to the curves. The narrowing of the voltammograms below 90.6 mV was seen regardless of the electrolyte's pH.

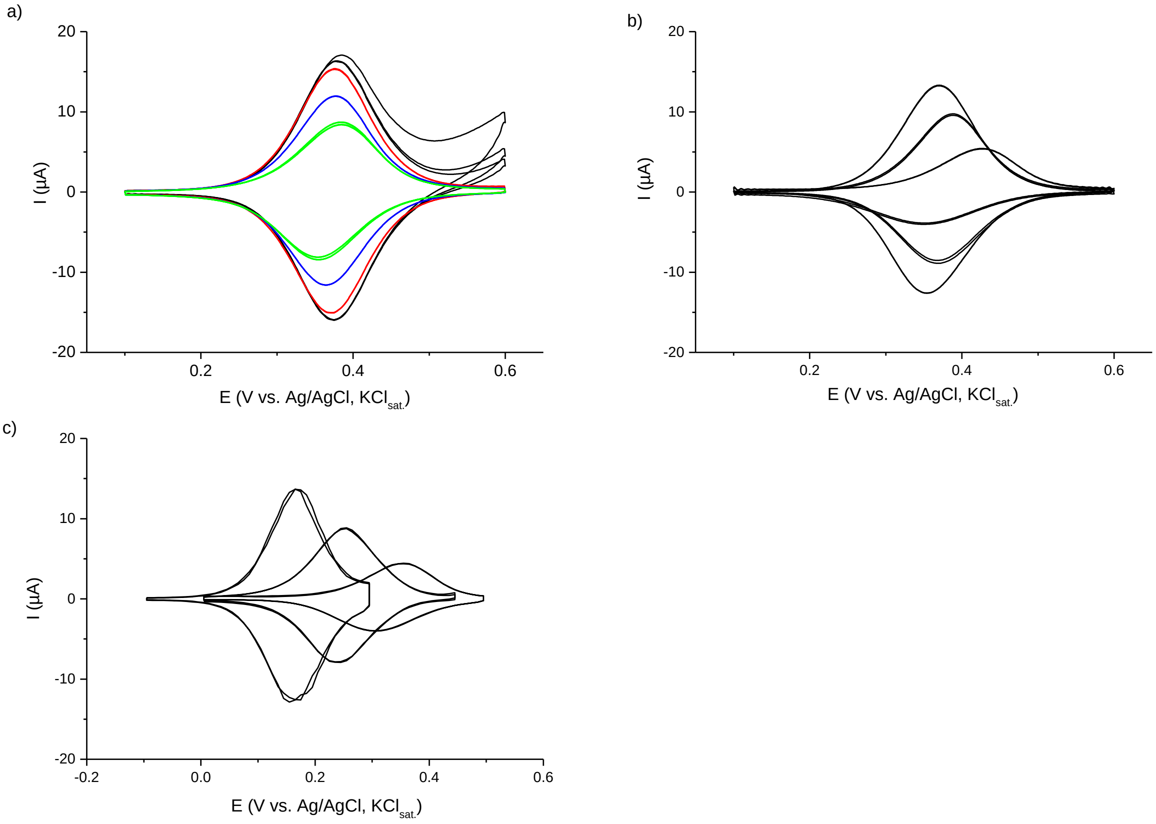

Supplementary Figure 6. Evolution of the voltametric shapes upon anodic treatment of highly-doped electrodes. Cyclic voltammograms (250 mV s^{-1}, 1.0 M $HClO_4$) for as-prepared and oxidized **S-2** samples. **(a)** The anodic treatment of samples prepared on highly-doped p-Si; as-prepared (black trace), and after consecutive anodic steps of 0.7 V / 20 s (red trace), 0.8 V / 120 s (blue trace) and 0.9 V / 30 s (green trace). **(b-c)** Data for highly-doped n-Si before and after consecutive anodic steps of 0.5 V / 30 s either under dark **(b)** or under illumination **(c)**. For all of these highly-doped samples **(a-c)** the anodic treatment of the sample has no effect on reducing fwhm's below 90.6 mV.

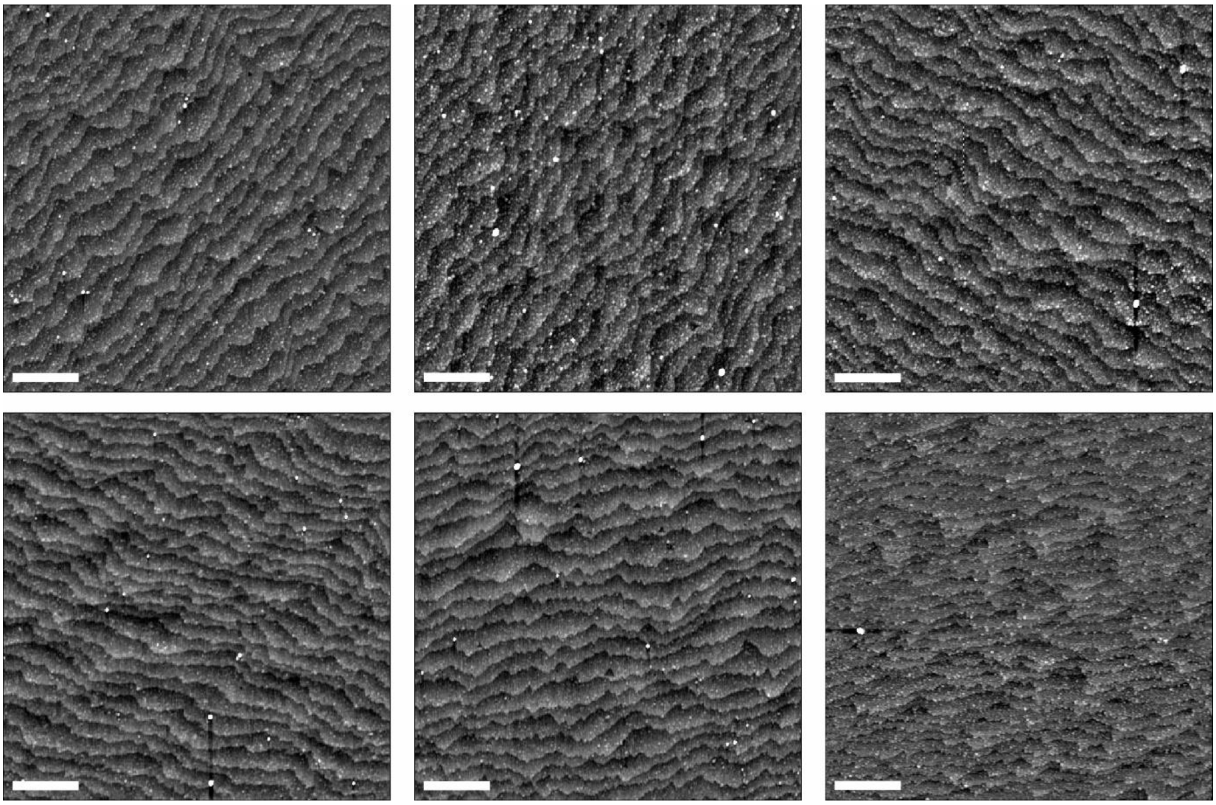

Supplementary Figure 7. **Tapping mode AFM images (3 × 3 μm) for Si(111) S-2 samples.** Data for as-prepared samples (left column), after applying a potential step leading to narrow peaks in cyclic voltammetry (central column) and data for oxidized samples where the narrow waves are lost (right column). It is a possibility that the observed rounded protrusion are oxide clusters. The number of protrusions systematically increases after anodic treatments. Anodization of the samples also results in an increase of the surface roughness R_q on the terraces (R_q is 0.14 ± 0.01 nm (R_{tm} = 17 ± 0.05 nm), before electrochemistry and 0.20 ± 0.02 nm (R_{tm} = 0.25 ± 0.08 nm), after the anodic treatment). The scale bar is 500 nm and the images high is normalized to 1.4 nm for comparison purposes.

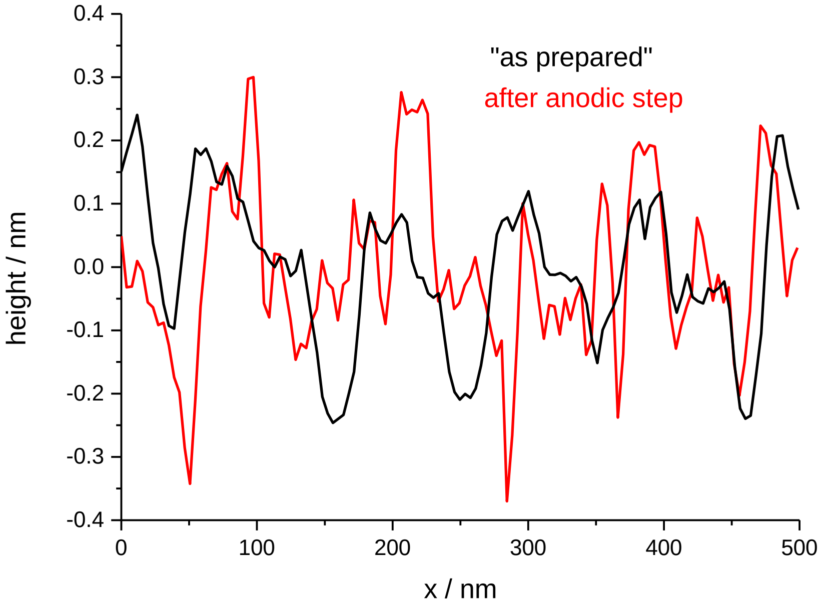

Supplementary Figure 8. Typical AFM profile along the terraces of S-2 samples on n-type Si(111). The black trace is for an as-prepared sample and the red trace is measured on a sample after having applied an anodic step.

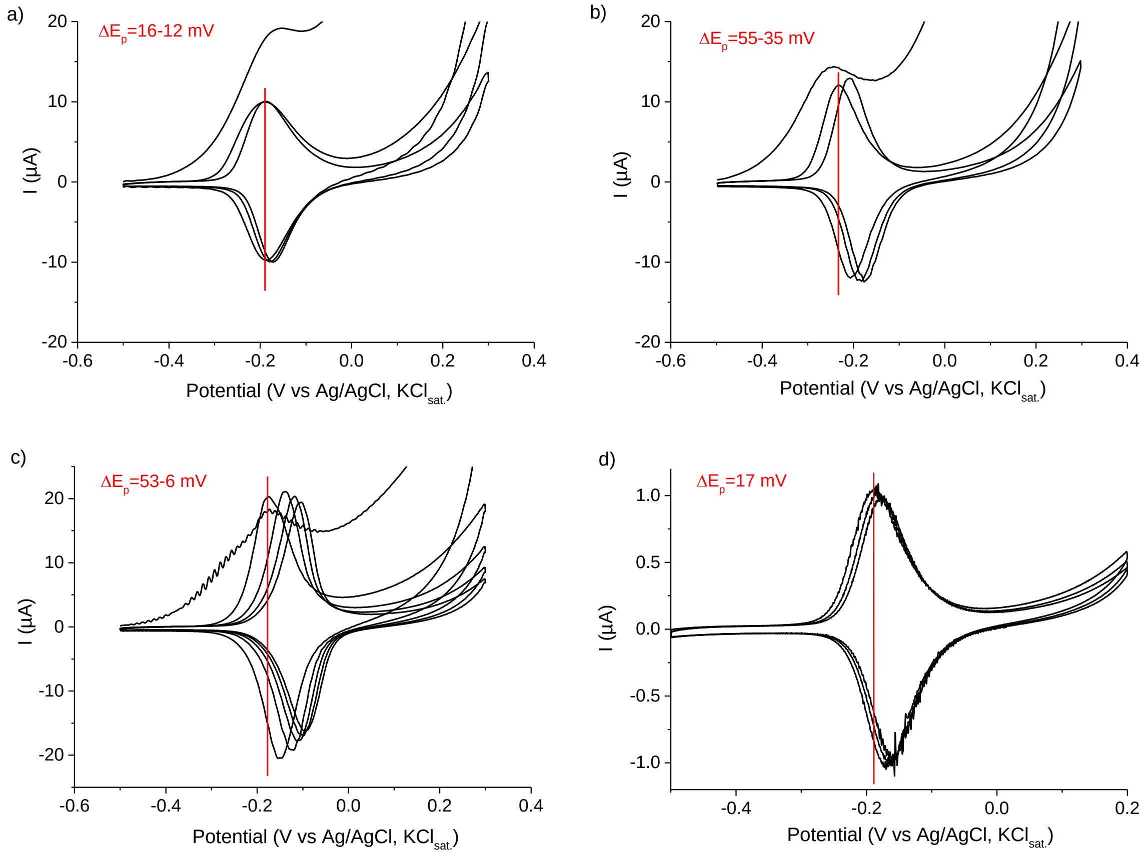

Supplementary Figure 9. **Examples of cyclic voltammograms with inverted peaks for n-type Si(111) S-2 samples in 1.0 M NaClO$_4$.** Samples showed inverted peaks only if the hydrosilylation reaction time to prepare **S-1** samples was lowered from 2 h to 2 or 10 min (ΔE_p in figure is equal to -(E_{pa} –E_{pc}) and was between 6 and 55 mV, see also Fig. 2c in the main text). The peak potential inversion is observed for all of the 2 min samples (panels **(a)** and **(b)**) and in 50% of the 10 min samples (panel **(c)**). In these samples peak potential inversion did not require an oxidative pre-treatment. **(d)** An oxidative pre-treatment was however required to lead to the peak potential inversion in 50% of the 10 min samples, and for these samples peak potential inversion was only apparent at very low scan rates (< 100 mV s^{-1}). For all of these samples (n-type) the peal potential inversion was short-lived, presumably because only a small fraction of the silicon is protected by the grafted diyne molecules (see Supplementary Fig. 11 and Supplementary Methods), and this instability did not allow further analysis/modelling (Model 3, Supplementary Note 6) of the data. Inverted peak potentials voltammograms on a-Si (Fig. 5 in main text) are significantly more stable and where used for fitting purposes. For example, data in panel **(c)** the peak inversion decreased rapidly from 53 mV in the 1st cycle to 6 mV in the 5th cycle. All cyclic voltammograms shown here were recorded at a scan rate of 250 mV s^{-1}, except for **(d)** where the scan rate is 25 mV s^{-1}. In **(d)** the sample's oxidation decreases the rate constant and at higher scan rates the peak potential inversion is shielded by the slow kinetics (see Fig. 4 in main text).

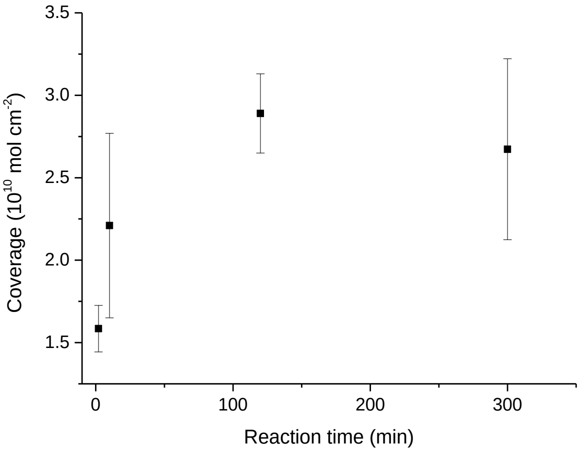

Supplementary Figure 10. **Electrochemically-determined surface coverage (mol cm^{-2}) of ferrocene units in lowly-doped n-type Si(111) S-2 samples.** The reaction time entry (x-axis) refers to the hydrosilylation reaction time used to prepare **S-1** samples and it was varied between 2 min and 300 min (2, 10, 120 and 300 min). For the monolayer works (**S-1**) in this work we used blue radiation (365 nm) to assist the hydrosilylation on Si-H of 1,8-nonadiyne **1**. Reaction times greater than 2 h do not yield coverages above 65% of a full monolayer (**S-2**). Comparable coverages are reported in literature[1] for hydrosilylation reactions of 1-alkynes under irradiation of $\lambda <$ 355 nm. It is not rigorous to state that the hydrosilyation leading to **S-1** is complete just by measuring values of Γ in samples of **S-2**, but on the other hand it is correct to say that hydrosilylation times shorter than 120 min are not leading to maximum coverages of diyne **1** (see also Supplementary Figure 12 and Supplementary Methods).

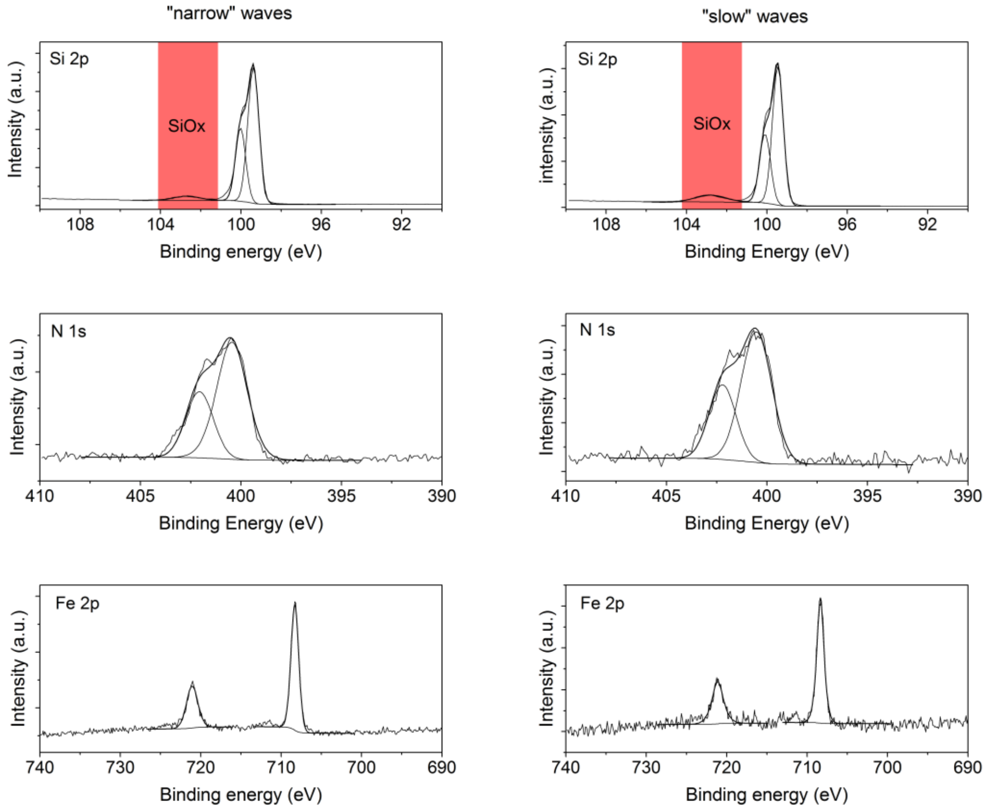

Supplementary Figure 11. **XPS spectrographs of S-2 samples after the anodic electrochemical experiments of Fig. 3 of the main text.** The label "narrow" waves for the XPS data presented on the left column is a short hand notation for **S-2** samples that were treated by a short anodic potential step (0.3 V, 2 min), a step that consistently leads to narrow waves of *ca*. 50-60 mV fwhm's. The relative peak area ratios of the SiO_x:Si 2p fitted emissions is 0.05 and the refined Fe:N ratio is close to unity. XPS narrow scans labelled as "slow" waves (column on the right) are representative spectral data for extensively oxidized **S-2** electrodes (consecutive steps of 0.3, 0.5 and 0.7 V, 50 s each step). The SiO_x:Si 2p ratio increases to 0.09 and the Fe:N ratio is close to 0.7.

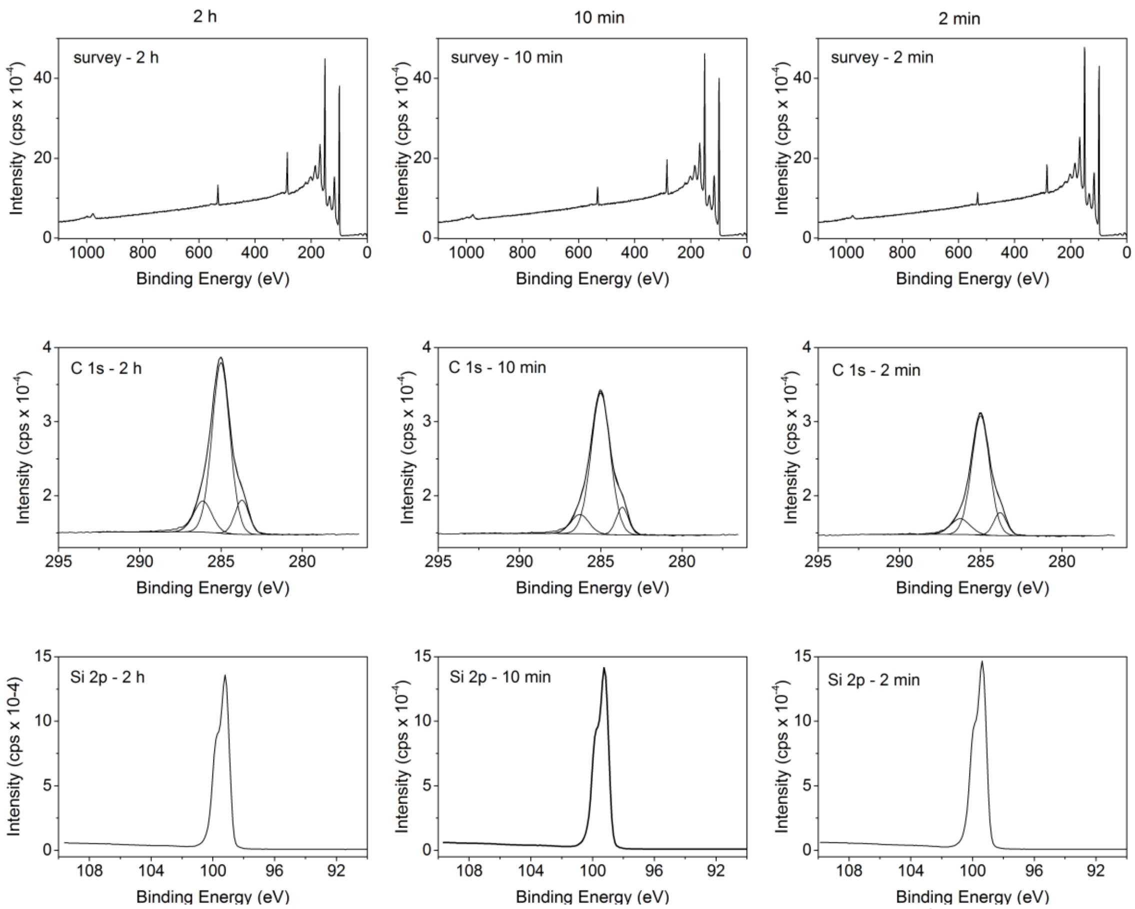

Supplementary Figure 12. **Representative survey, C 1s and Si 2p high-resolution XPS data for the hydrosilylation under 365 nm radiation of 1,8-nonadiyne 1 samples on Si(111)-H surfaces (S-1).** Hydrosilylation reaction times were: 2 h, left column; 10 min, central column; and 2 min, right column. The approximate ratio of carbon chains to silicon surface atoms – i.e. the fractional monolayer (ML) coverage (N_{ML}/N_{Si}) - was estimated form the Si 2p and C 1s signals (see below) and are 0.32 (2 h), 0.25 (10 min), 0.20 (2 min). To attempt a comparison with UV-assisted hydrosilylation reactions (e.g. Hg lamps, 254 nm) may turn to be speculative, nevertheless it is of some interest to note that the N_{ML}/N_{Si} numbers reported by Cicero and co-workers[2] for reactions of phenylacetylene samples on Si(111)-H are 0.36 and 0.20 (2 h and 12 min hydrosilylation reaction time) and those for samples of 1-octyne are 0.45 and 0.40 (2 h and 12 min).

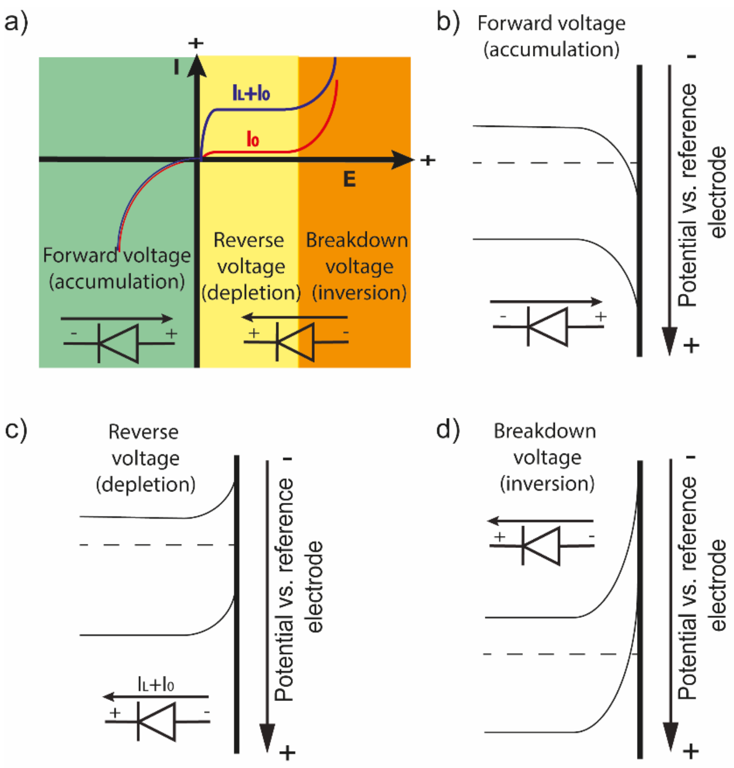

Supplementary Figure 13. **Schemes depicting the flow of currents and the band-bending for different potential regimes in an n-type semiconductor photoanode behaving like an ideal diode.** There are three different regimes depending on the applied potential and these are shown in **(a)** under illumination (blue curve) and when shielded from the light (red curve). High cathodic currents flow when in accumulation (green background), and only a small leakage anodic current flows (I_0) when in depletion at positive overpotentials (yellow background). When illuminated in the depletion region the anodic current is increased by a quantity indicated as I_L. When applying large positive overpotentials the anodic current increases again, regardless if under illumination or not (inversion, orange background). In **(b-d)** the silicon band diagrams are shown for each regime with the respective diode orientation and direction of the electron flow. Scheme is not to scale and the IUPAC convention is used for the currents, i.e. the arrows represent the flow of electrons and the positive current is anodic.

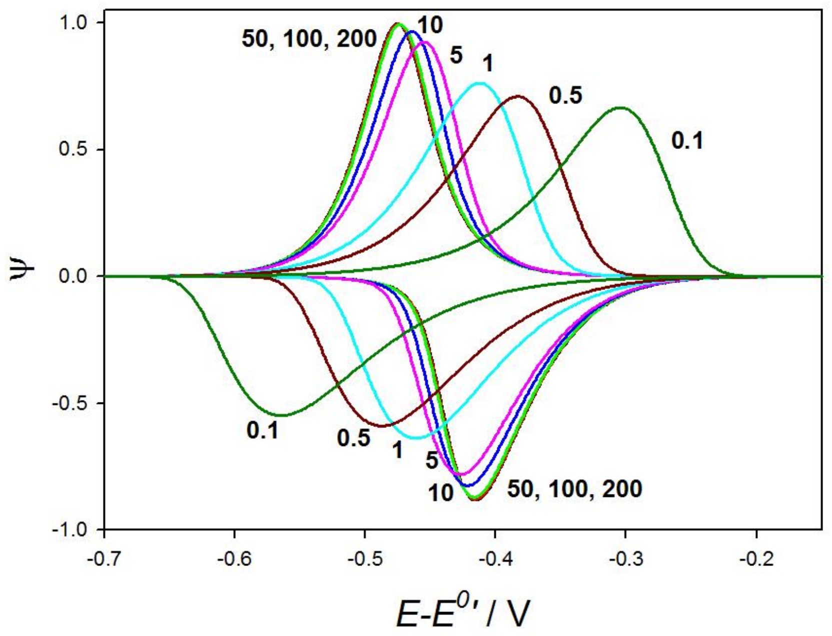

Supplementary Figure 14. Cyclic voltammetry for a semiconductor/electrolyte interface under finite kinetics limit. CV curves calculated from (Supplementary Equation 47) and (Supplementary Equation 53) for different values of the rate constant $\boldsymbol{k_{et}}$ indicated in the figure (in s^{-1}) as labels to the curves. The other simulations parameters are $\boldsymbol{\alpha = 0.5}$, $\boldsymbol{I_0 = 10^{-6}}$ μA, $\boldsymbol{I_L(\text{direct scan}) = 100}$ μA, $\boldsymbol{I_L(\text{reverse scan}) = 2}$ μA (diode behaves as photoanode), D = 1, $\boldsymbol{Q_F = 6}$ μC, $\boldsymbol{v = 25}$ mV $\mathbf{s^{-1}}$. Interaction parameters are G = 0.5, s = -0.2 and y = 1.0.

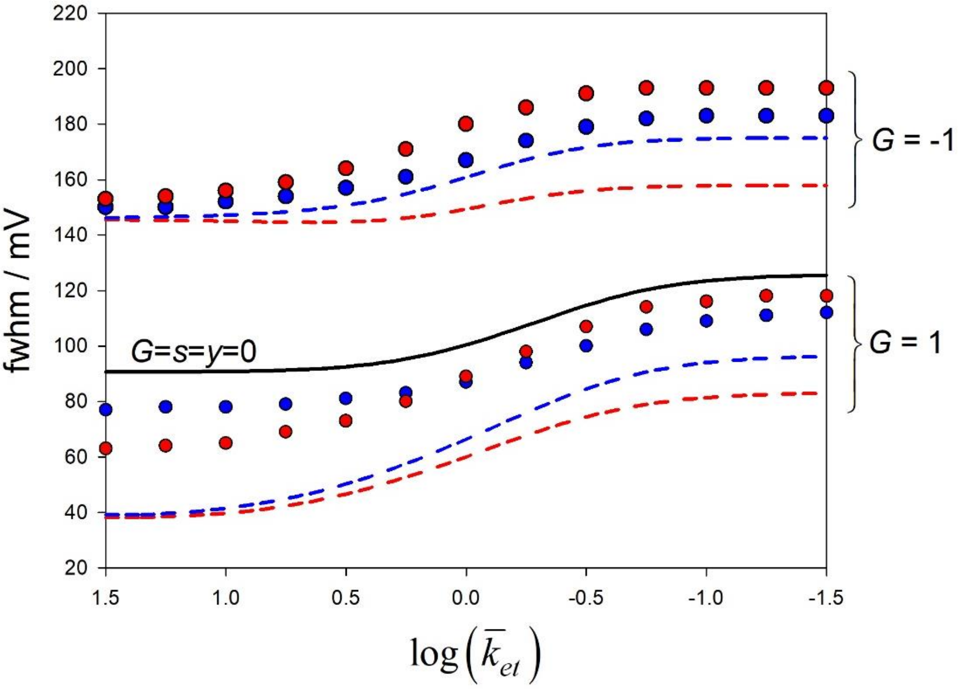

Supplementary Figure 15. Variation of the voltammetry fwhm's (in mV) in response to changes of the logarithm of the dimensionless rate constant $\bar{k}_{et}$. $\alpha = 0.5$. Black line corresponds to model 1 (absence of interactions and diode effects, (Supplementary Equation 11)). Dashed lines correspond to Model 2 (interactions), and have been obtained from (Supplementary Equation 24) by using the following values: *s*=-0.5; *y*=0.5; *G* value indicated in the curves. Symbols correspond to Model 3 (interactions and diode effects), and have been calculated from (Supplementary Equation 53) by considering a photoanode behavior with $I_0 = 10^{-6}$ μA; $I_{La} = 100$ μA; $I_{Lc} = 50$ μA; *s*=-0.5; *y*=0.5; *G* value indicated in the curves. Blue lines / symbols refer to the direct (anodic) scan and red lines / symbols to the reverse (cathodic) one. Voltammetry fwhm is a parameter very sensitive to the nature of the charge-transfer process. When neither interactions nor diode effects are considered (ideal behavior), fwhm's increase when the charge-transfer becomes slower until it reaches a constant value of 125 mV for fully irreversible processes ($\alpha = 0.5$). It can be also observed that in this case anodic and cathodic peaks present the same fwhm value. This symmetry is broken when interactions are considered (lines). In the case of predominant attractive interactions (*G* = 1), the fwhm is always below the ideal value, whereas for the contrary case (*G* = -1), fwhm is always higher than ideal values. In the case of interactions and diode effects (symbols), the situation can become even more complex, with a stronger asymmetric influence in the peaks for the direct and reverse scans (see curves for *G* = 1). Thus, the determination of the different parameters involved in the process from the analysis of fwhm alone will give rise to significant errors and a detailed analysis of the different parameters dominating the response in terms of the scan rate is therefore required.

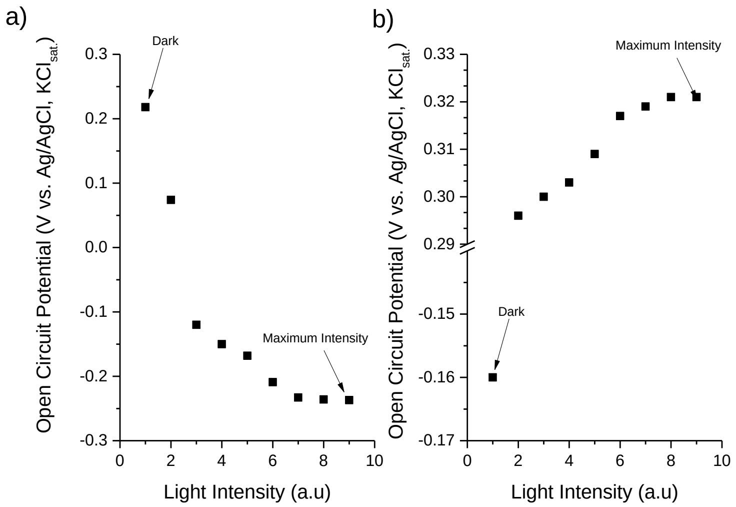

Supplementary Figure 16. **Shifts in the open-circuit potential with changes to the illumination intensity for S-2 samples on either (a) Si(111) n-type or (b) a-Si substrates.** Electrolyte is 1.0 M $HClO_4$. The trend in the plots shows that Si(111) samples behaves like photoanodes, as expected due to the n-type doping, while and the intrinsic a-Si film acts as a photocathode as a consequence of its back contact with a p-type Si(100) wafer.

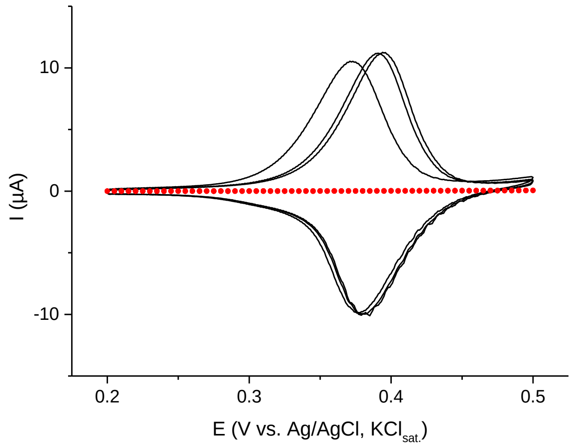

Supplementary Figure 17. **Samples on amorphous silicon**. Cyclic voltammograms (100 mV s^{-1}) for a-Si **S-2** samples under illumination (continuous black line) and dark (red symbols) conditions. Electrolyte is aqueous 1.0 M $HClO_4$.

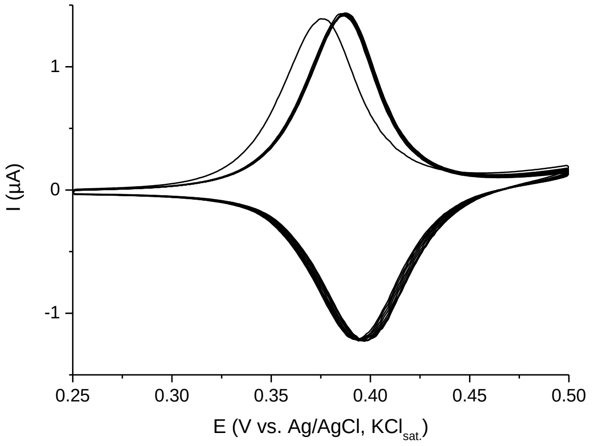

Supplementary Figure 18. Stability. Cyclic voltammetry (10 mV s^{-1}) in 1.0 M $HClO_4$ for a-Si **S-2** samples showing no measurable changes to peak positions and current intensity with continuous cycling.

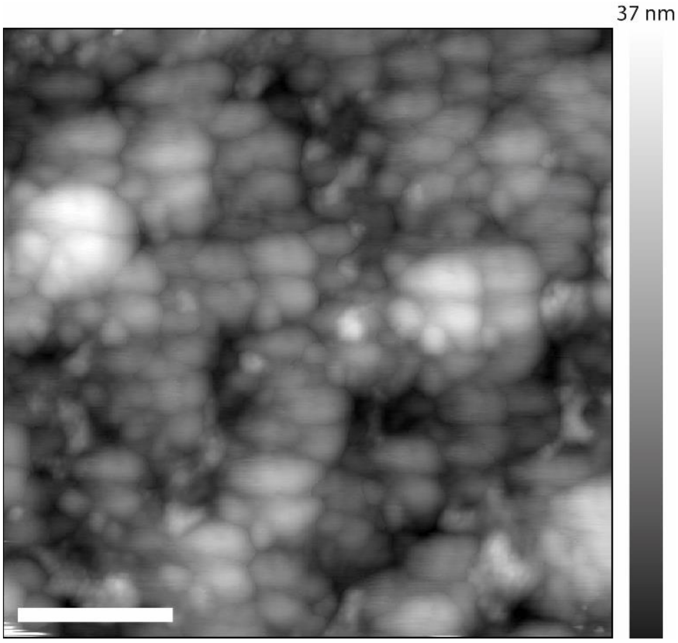

Supplementary Figure 19. **Representative AFM micrograph for a-Si S-2 sample.** The sample roughness is large, with an estimated R_q value of 4.2 nm (R_{tm} = 4.7 ± 0.52 nm). The scale bar is 500 nm.

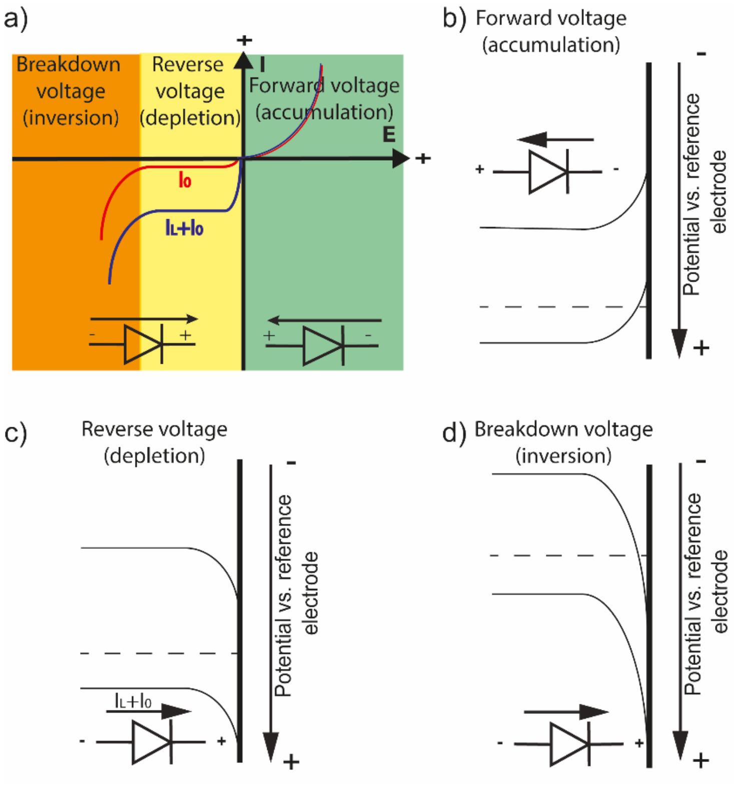

Supplementary Figure 20. Schemes depicting the flow of currents and the band-bending for different potential regimes in a p-type semiconductor photocathode behaving like an ideal diode. Three different regimes as a function of the applied potential and are shown in **(a)**, under illumination (blue solid curve) and when shielded from the light (red solid curve). High anodic currents flow when in accumulation (green background), and only a small leakage cathodic current flows (I_0) when in depletion at negative overpotentials (yellow background). When illuminated in the depletion region the cathodic current is increased (photogenerated current, I_L). When applying a high negative overpotential the cathodic current increases again regardless if under illumination or not (inversion, orange background). In **(b-d)** the corresponding silicon band-diagrams are shown for each regime with the respective diode orientation and direction of the electron flow. Scheme is not to scale and the IUPAC convention is used for the currents (arrows represent the flow of electrons and the anodic current is positive).

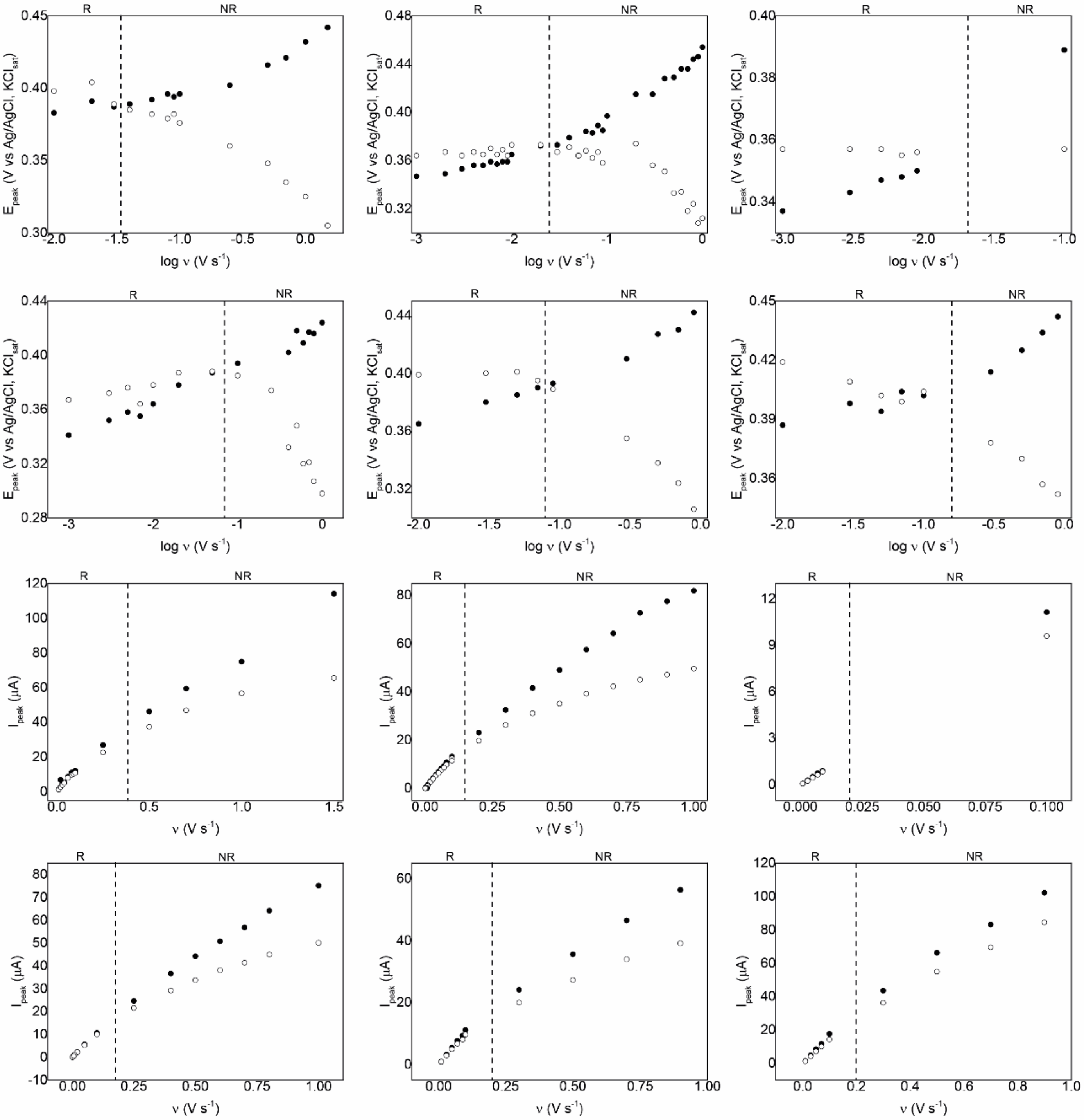

Supplementary Figure 21. Kinetic regions for the voltammetry as a function of the scan rate. Evolution of the experimental peak potentials (E_{peak}, top two rows) and currents (I_{peak}, bottom two rows) as a function of voltage sweep rate (ν) in the cyclic voltammetry of **S-2** samples on a-Si. Black and white symbols correspond to the anodic and cathodic data, respectively. Reversible (fast kinetics, R) and non-reversible (finite slow kinetics, NR) zones are qualitatively indicated in figure. Lines are a guide to the eye only.

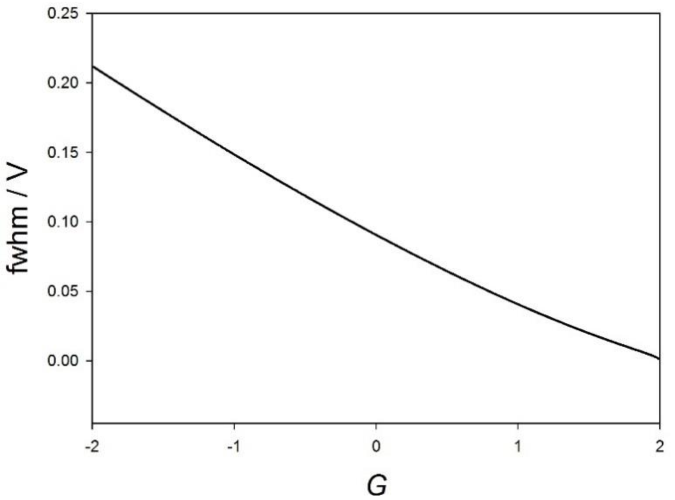

Supplementary Figure 22. **Fwhm and *G*.** Variation of the voltammetry fwhm's (in V) in response to changes of the interaction parameter *G* as calculated from (Supplementary Equation 20).

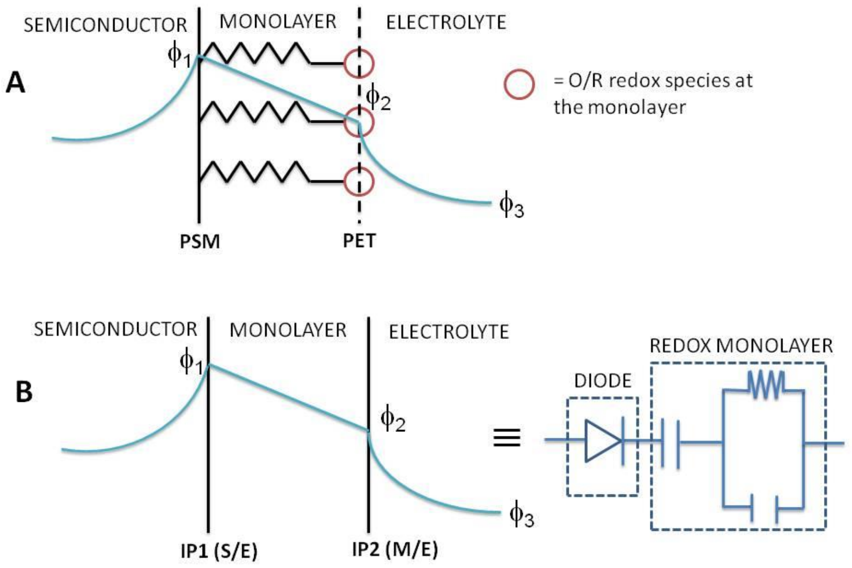

Supplementary Figure 23. Semiconductor/monolayer/electrolyte as two polarizable interfaces in series. A Schematic view of the semiconductor/monolayer/electrolyte. PSM and PET are the short hand notations to indicate the semiconductor/monolayer plane and the plane of electron transfer at the redox centre of the monolayer, respectively. Blue line depicts the evolution of electric potential; its value at specific locations is indicated as **1** for semiconductor/monolayer interface, **2** for the monolayer/solution interface, and **3** is the bulk of the solution. **B (Left)** Simplified model for the interface given in A as a series arrangement of two polarizable interfaces. **(Right)** Tentative equivalent circuit for the system. In this scheme the diode has been considered as a photocathode (e.g. a-Si samples).

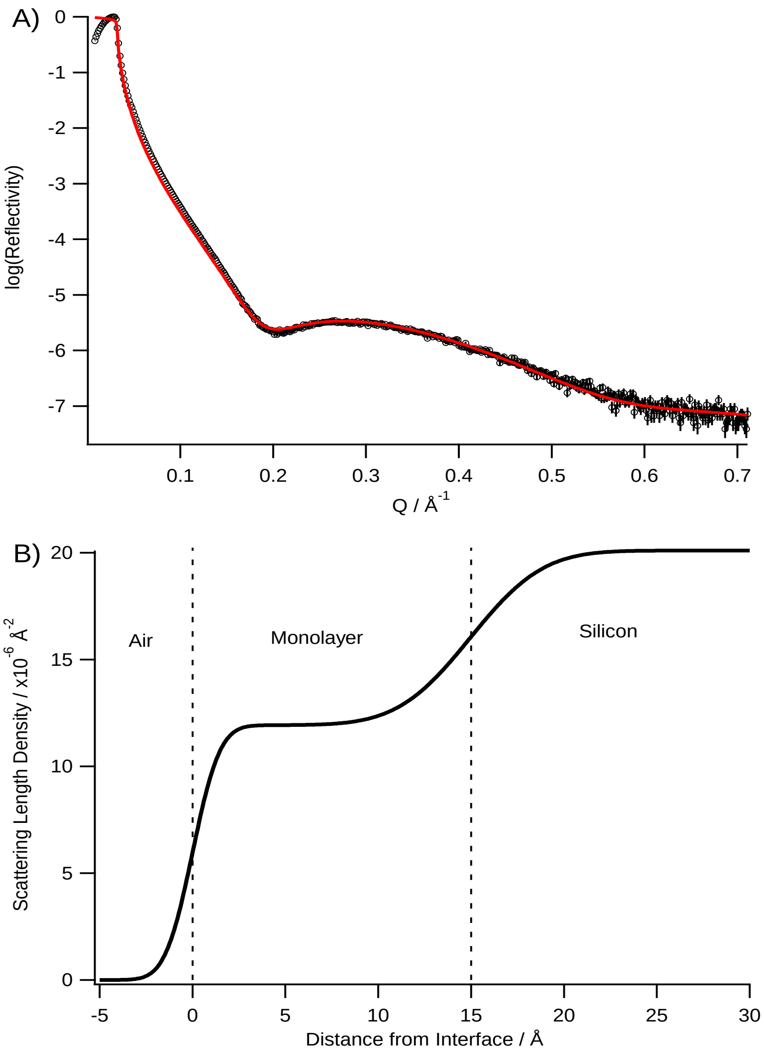

Supplementary Figure 24. X-ray reflectometry. ***Experimental monolayer thickness, electron density and roughness***. A) X-ray reflectivity profile of the **S-2** monolayer system on Si(111). The points with error bars are the collected data and the red line is the fit to the data. B) Real-space SLD profile of the monolayer systems with distance 0 Å set as the interface between air and the monolayer. The vertical dashed lines show the boundary between the air/monolayer and monolayer/silicon interfaces.

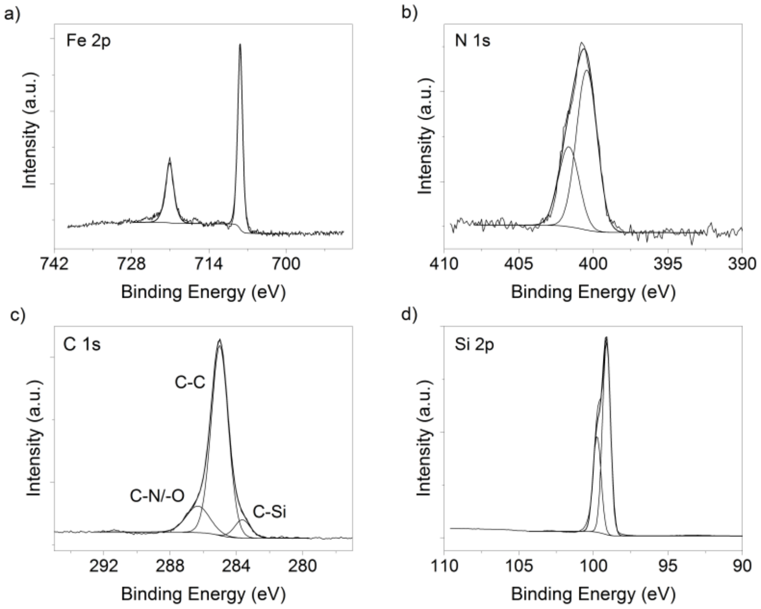

Supplementary Figure 25. XPS spectrographs for as-prepared ferrocene-derivatized Si(111) surfaces (S-2). Samples of **S-2** were obtained via CuAAC reactions between azidomethylferrocene **2** and samples of **S-1** (lowly-doped, n-type, 2 h hydrosilylation reaction time). (a) Narrow scan of the Fe 2p region showing two spin–orbit-split components 3/2 and 1/2, (b) N 1s region with no evidence of unreacted azido groups (ca. 404 eV), (c) high resolution scans of the C 1s region, and (d) high-resolution scan for the Si 2p region. Absent from the spectra is a silica-related emission (102–104 eV).

Supplementary Table 1. ***Results from fits to X-ray reflectometry data for the S-2 Si(111) samples modified with 1,8-nonadiyne-ferrocene.*** NOTE: SLD (scattering length density) for X-rays is obtained by multiplying the electron density ($e^-/Å^3$) of the material by the factor 2.82×10^{-5} Å. *The SLD for air and silicon are known and were fixed parameters.

Layer	Thickness / Å	SLD / $\times 10^{-6}$ Å^{-2}	Roughness / Å
Air	-	0.0 *	-
Organic monolayer	14.9 ± 0.2	11.9 ± 0.1	1.2 ± 0.1
Silicon substrate	-	20.1 *	3.1 ± 0.1

Supplementary Note 1: The Langmuir model for a single charge-transfer (*Model 1*)

Fast kinetics limit

This model describes the analytic current-voltage relationship for a reversible redox process, $R \rightleftharpoons O + ne^-$, and assumes: i) O and R (the oxidized and reduced electroactive species, respectively) to be strongly adsorbed - nor adsorption or desorption occur, ii) molecules of O and R occupy equals areas on the electrode surface, iii) the local potential at the reaction site is independent of the extent of charge-transfer and iv) interactions between adsorbed species can be neglected (i. e. adsorption coefficients for O and R are potential independent). The relationship between the surface coverage of species O and R and the electrochemical potential $\bar{\mu}$ defines the Langmuir's isotherm

The relationship between the surface coverage of species O and R and the electrochemical potential $\bar{\mu}$ defines the Langmuir's isotherm

$$\left.\begin{aligned} \exp\left(\frac{\bar{\mu}_\mathrm{O}}{RT}\right) &= \frac{f_\mathrm{O}}{1-f_\mathrm{O}-f_\mathrm{R}} \\ \exp\left(\frac{\bar{\mu}_\mathrm{R}}{RT}\right) &= \frac{f_\mathrm{R}}{1-f_\mathrm{O}-f_\mathrm{R}} \end{aligned}\right\} \qquad \text{(Supplementary Equation 1)}$$

where $f_\mathrm{i} = \Gamma_\mathrm{i} / \Gamma_\mathrm{E}$, Γ_E is the maximum surface coverage, $\bar{\mu}_i = \mu + z_i F\phi^\varsigma = \mu_i^{0,\varsigma} + RT\ln a_i + z_i F\phi^\varsigma$, and ϕ^ς is the Galvani potential at the phase ς. Γ_T is the total surface excess which remains constant during the experiment.

By dividing the two equations appearing for O and R in (Supplementary Equation 1) and taking into account that $(\bar{\mu}_\mathrm{O} - \bar{\mu}_\mathrm{R})/RT = F\left(E - E^{0'}\right)/RT$, with $E^{0'}$ being the formal potential of process (I), a potential-coverage relationship formally identical to Nernst's equation is obtained,

$$E - E^{0'} = \frac{RT}{F}\ln\left(\frac{f_\mathrm{O}}{f_\mathrm{R}}\right) \qquad \text{(Supplementary Equation 2)}$$

This implies that the electron transfer process is reversible and is a good approximation for fast electron transfer and slow scan rates (*vide infra*). The current is given by

$$I = -nFA\frac{d\Gamma_r}{dt} = -nQ_F\frac{df_r}{dt} \qquad \text{(Supplementary Equation 3)}$$

$$Q_F = FA\Gamma_T \qquad \text{(Supplementary Equation 4)}$$

$$f_i = \frac{\Gamma_i}{\Gamma_T}, \qquad i = \text{O, R} \qquad \text{(Supplementary Equation 5)}$$

where a positive sign of the current corresponds to the anodic process, A is the area of the electrode and

$$\Gamma_T = \Gamma_o + \Gamma_r \qquad \text{(Supplementary Equation 6)}$$

is the total excess. The applied potential E changes over time t as

$$\left.\begin{aligned} E(t) &= E_i + vt && \text{for } t \le t_{inv} \\ E(t) &= E_f - v(t - t_{inv}) && \text{for } t > t_{inv} \end{aligned}\right\} \qquad \text{(Supplementary Equation 7)}$$

being E_i, E_f and t_{inv} the initial and final applied potentials, and the time at which the scan is inverted. The voltage scan rate is indicated as v.

Then, an analytic *I-E* expression can be obtained from the Nernst equation, the Langmuir isotherm and (Supplementary Equation 1) and (Supplementary Equation 5):

$$I = \pm \frac{1}{H} \frac{J}{(1+J)^2} \qquad \text{(Supplementary Equation 8)}$$

where the upper / lower sign corresponds to the direct / reverse scan. Moreover

$$H = \frac{RT}{n^2 F v} \frac{1}{Q_F} \qquad \text{(Supplementary Equation 9)}$$

and

$$J = \exp\left(\frac{nF}{RT} \left(E(t) - E^{0'} \right) \right) \qquad \text{(Supplementary Equation 10)}$$

where *n*, *F*, *R* and *T* have the common meaning (see glossary in Supplementary Note 8) and $E^{0'}$ is the formal potential.

Finite kinetics limit

The Langmuir model can be expanded to account for any degree of reversibility,[3] taking into account the finite electron transfer kinetics. This model was used for the calculation of rate transfer constants when diode and interactions are neglected. The model still holds assumptions i-iii). The *I-E* relationship is described by the Butler-Volmer equation,

$$I = \frac{\bar{k}_{et}}{H} \left[f_r J^{(1-\alpha)} - f_o J^{-\alpha} \right] \qquad \text{(Supplementary Equation 11)}$$

where

$$\bar{k}_{et} = \frac{k_{et}}{\left(\frac{nFv}{RT} \right)} \qquad \text{(Supplementary Equation 12)}$$

being k_{et} the rate constant at $E = E^{0'}$ and α the symmetry factor introduced in the Butler-Volmer equation. The potential dependence of Γ_o and Γ_r cannot be described with the Nernst equation, but can instead be obtained by combining (Supplementary Equation 3), (Supplementary Equation 6), (Supplementary Equation 7) and (Supplementary Equation 11):

$$\left. \begin{aligned} \frac{df_r}{d\eta} &= -\bar{k}_{et} f_r \left[J^{-\alpha} + J^{(1+\alpha)} \right] + \bar{k}_{et} J^{-\alpha} && \text{direct scan} \\ \frac{df_o}{d\eta} &= -\bar{k}_{et} f_o \left[J^{-\alpha} + J^{(1+\alpha)} \right] + \bar{k}_{et} J^{(1+\alpha)} && \text{reverse scan} \end{aligned} \right\} \qquad \text{(Supplementary Equation 13)}$$

with

$$\eta = \frac{nF}{RT} \left(E - E^{0'} \right) \qquad \text{(Supplementary Equation 14)}$$

By solving (Supplementary Equation 13), f_r and f_o can be obtained taking account (Supplementary Equation 6) and the *I-E* voltammogram is obtained by replacing the obtained values into (Supplementary Equation 11).

The reversibility is accounted by the value of m, which depends on the electron transfer kinetics and the sweep rate. For high values of $\bar{k}_{et}$ ($\bar{k}_{et} > 3$) the voltammograms show the same behavior as in the fast kinetic model.

Supplementary Note 2: The interaction model for a single charge-transfer (*Model 2*).

Fast kinetics limit

This model is similar to Model 1 (in Supplementary Note 1), but now the electrostatic interactions that push/pull the attached electroactive molecules are introduced through the Frumkin isotherm.[4] This model was used to simulate data presented in Fig. 2 of the main text. Assumptions i), ii) and iv) still applies, but iii) must to be corrected to consider the Frumkin isotherm that takes into account the interactions between the adsorbed electroactive species. It is usually assumed that all adsorbates on the surface lattice are randomly mixed and uncorrelated. As a result, the average interaction energy an adsorbate feels from the others is proportional to the coverage *f*. If we restrict to the nearest-neighbor interactions, an additional energy term should be added to the electrochemical potential we introduce interaction parameters a_o, a_r and a_{or} between O–O, R–R and O–R molecules, respectively, which are potential independent. Parameters a_o and a_r can be thought of as repulsions and a_{or} can be thought of as attractions. Under these conditions, the Frumkin isotherm for two adsorbates O and R is given by,

$$\left.\begin{aligned} \exp\left(\frac{\bar{\mu}_o}{RT}\right)\exp\left(2a_o f_o + 2a_{or} f_r\right) = \frac{f_o}{1-f_o-f_r} \\ \exp\left(\frac{\bar{\mu}_r}{RT}\right)\exp\left(2a_r f_r + 2a_{or} f_o\right) = \frac{f_r}{1-f_o-f_r} \end{aligned}\right\} \quad \text{(Supplementary Equation 15)}$$

Taking into account the above equation together with (Supplementary Equation 3), (Supplementary Equation 6) and, (Supplementary Equation 7), the f_r and f_o dependence with potential can be obtained,

$$E = E_p + \frac{RT}{nF} ln\left(\frac{1-f_r}{f_r}\right) + jG\left(2f_r - 1\right) \quad \text{(Supplementary Equation 16)}$$

where

$$E_p = E^{0\prime} - \frac{RT}{nF} ln\left(\frac{b_o}{b_r}\right) + \frac{RT}{nF} s \quad \text{(Supplementary Equation 17)}$$

$$G = a_o + a_r - 2a_{or} \quad \text{(Supplementary Equation 18)}$$

$$s = a_r - a_o \quad \text{(Supplementary Equation 19)}$$

being *j* the number of water molecules displaced by one molecule of O or R (that we will assume as equal to one), b_o and b_r the adsorption coefficients of species O and R, respectively, and the current dependence with f_r and f_o is given by:

$$I = \pm \frac{1}{H} \frac{f_r\left(1-f_r\right)}{1-2jGf_r\left(f_r\right)} \quad \text{(Supplementary Equation 20)}$$

The peak current can be obtained from the following,

$$I_p = \pm \frac{1}{H} \frac{1}{4-2G}$$ (Supplementary Equation 21)

The current-potential response can be obtained by combining the non-explicit expression of the current given by (Supplementary Equation 20) and the potential-coverage relationship given by (Supplementary Equation 16). Parameter *G*, as specified in (Supplementary Equation 18), does gather the interaction parameters, thus it accounts for the overall interactions sensed by the electroactive adsorbed molecules and it is responsible for changes to the full width half maximums (fwhm's) in the cyclic voltammogram waves (see for example Fig. 2 in the main text). Changes to parameter *s* are reflected on shifts to the position of the voltammetry peaks through (Supplementary Equation 17).

More recently, Aleveque *et al.* have developed extended version of Laviron's original model for general lateral interactions,[5] and they have provide an approximate explicit equation for the current in terms of the potential,

$$I = \pm \frac{1}{H} \frac{\xi}{(1+\xi)^2 - 2G\xi}$$ (Supplementary Equation 22)

where

$$\xi = \exp\left(\frac{nF}{RT}\left(E - E_p\right) + \frac{2G}{1 + exp\left(-\frac{nF\left(E - E_p\right)}{(1-0.4G)RT} \right)} \right)$$ (Supplementary Equation 23)

Finite kinetics limit

Under finite kinetics the coverage potential relationship given by (Supplementary Equation 16) is not valid and a more general approach must be followed. Laviron deduced an expression for the current-potential relationship in terms of the interaction coefficients a_o, a_r and a_{or} for any degree of reversibility of the charge-transfer reaction by considering that the activation free energies of direct and reverse reactions are affected by the presence of interactions in a linear way.[6] We can re-write the expression reported by Laviron (Eq. (3) in [6]) in terms of linear combinations of the interaction parameters *G*, *s* and *y* in the way

$$I = \frac{1}{nQ_F} k_{red} \left\{ f_r e^{\eta} e^{-(y-G) - f_r(G+s)} - (1-f_r) e^{-(y-s)+f_r(G-s)} \right\}$$ (Supplementary Equation 24)

where parameters *G* and *s* are given by (Supplementary Equation 18) and (Supplementary Equation 19), and

$$y = a_o + a_r$$ (Supplementary Equation 25)

The expression of k_{red} depends on the particular kinetic model chosen for describing the charge-transfer reaction. Thus, if we consider the Butler-Volmer approach,

$$k_{red} = k_{et} e^{-\alpha\eta}$$ (Supplementary Equation 26)

with k_{et} and α being the conditional rate constant (*i. e.*, the value of the rate constant for electro-reduction or electro-oxidation at $E = E^{0\prime}$), and the charge-transfer coefficient, respectively. If another model, e. g., Marcus-Hush, is chosen, the expression of k_{red} is more complex (see for example [7]).

As in the case of fast kinetics, (Supplementary Equation 24) **does not** provide a direct relationship between the current and potential since the expression of f_r is unknown. Thus, a numerical procedure must be implemented in order to know the value of the surface coverage at a given potential.

Supplementary Note 3

Replacing 1,8-nonadiyne with 1,6-heptadiyne has the effect of shortening the ferrocene-to-silicon distance by ca. 2 Å. The XRR-determined monolayer thickness drops from 15.0 Å to 13.3 Å and the fwhm drop to ca. 80 mV. However, the effect is very short-lived and a systematic change in voltage sweep rates is accompanied by rapid loss of surface coverage.

Supplementary Note 4: XRR methods and data

Structural parameters for the monolayer were refined in MOTOFIT reflectometry analysis software.[8] The monolayer is conceptualised as a layered system with each layer defined by its thickness, X-ray scattering length density (SLD), and interfacial roughness. MOTOFIT utilises the Abeles matrix formalisation to calculate the specular reflectivity from a stratified layer system.[8] In this method the system is split into a series of layers and the incident radiation is refracted by each layer. The value of the wave vector (k) in layer n is given by:

$$k_n = \sqrt{k_0^2 - 4\pi\left(\rho_n - \rho_0\right)} \qquad \text{(Supplementary Equation 27)}$$

Where $k_0 = Q/2$ and ρ is the SLD of the layer. The Fresnel reflection coefficient ($r_{n,n+1}$) between layers n and $n+1$ is described by:

$$r_{n,n+1} = \frac{k_n - k_{n+1}}{k_n + k_{n+1}} exp\left(-2k_n k_{n+1}\sigma_{n,n+1}^2\right) \qquad \text{(Supplementary Equation 28)}$$

The term after the exponential function in the above equation is a Gaussian error function to account for the roughness between each interface.[9,10] The Fresnel reflection coefficients along with phase factors are used to calculate a characteristic matrix for each layer, the product of which is used to calculate the reflectivity. A least-square fitting routine is used to minimise χ^2 values between observed and calculated reflectivity using a genetic algorithm. The fitting of the reflectometry profile yields information on the SLD profile normal to the surface. The SLD can be considered as an X-ray refractive index and is a function of the chemical composition of the material according to:

$$SLD = \frac{\sum_{i=1}^{n} Z_i r_e}{V_m} \qquad \text{(Supplementary Equation 29)}$$

Where Z_i is the atomic number of the ith atom, r_e is the Bohr electron radius (2.818×10^{-15} m), and V_m is the molecular volume determined to be 375 Å^3 using the web tool Molinspiration (http://www.molinspiration.com/). From this the theoretical SLD of a complete monolayer is determined to be 14.2×10^{-6} Å^{-2}. By comparing the theoretical SLD to the fitted SLD of the monolayer the volume fraction (φ) of the monolayer can be determined as:

$$\varphi = \frac{SLD_{fitted}}{SLD_{theoretical}} \qquad \text{(Supplementary Equation 30)}$$

Using the volume fraction and other parameters the surface excess (Γ) in mol cm^{-2} can be calculated as follows:

$$\Gamma = \frac{\varphi \tau 10^{16}}{V_m N_A} \qquad \text{(Supplementary Equation 31)}$$

Where τ is the monolayer thickness and N_A is Avogadro's constant.

Measuring the X-ray reflectometry of the monolayer on silicon under ambient conditions showed a single broad fringe approximately $0.2 < Q < 0.6$ Å^{-1} indicating that a well formed monolayer had been deposited onto the silicon substrate (Supplementary Figure 24A). The best fit to the X-ray reflectometry data was using a model where the monolayer system is defined as a single layer (red line in Supplementary Figure 24A). The total thickness of the monolayer was determined to be 14.9 Å (Supplementary Table 1) which is consistent with previous monolayer systems that utilise the same click chemistry method for fabrication. The fitted SLD was found to be 11.9×10^{-6} Å^{-2} and this corresponds to a surface excess of 5.56×10^{-10} mol cm^{-2} which is consistent with the determination of surface coverage from the electrochemical data. The system consists of two interfaces at the air/monolayer interface and monolayer/silicon interface each with a roughness of 1.2 Å and 3.1 Å respectively (Supplementary Table 1 and Supplementary Figure 24B). The X-ray reflectometer was set up to illuminate a relatively large area of 10 × 10 mm and therefore provides a global view of roughness compared to AFM which sampled over a much smaller area of 2 × 2 μm providing a localised view of roughness determined to be 2.0 Å . The roughness values between the two independent techniques are consistent showing that the sample area chosen for AFM is typical across the larger area.

Supplementary Note 5

The term "peak inversion" refers here to the relative position of peak potentials in experimental voltammograms for single-electron transfer reactions at semiconductors. As discussed here, this peculiar inversion is electrostatic in origin. It should not be confused with the peak inversion previously reported in the literature of metallic electrodes which refers to an inversion in the sign of the current, e.g. cathodic currents for an anodic scan and due to the presence of adsorption steps in multi-electron transfer reactions and/or formation of new phases at the electrode surface. See for example [11] or [12].

Supplementary Note 6: The diode model with Frumkin interactions and finite kinetics (*Model 3*).

We developed a model to account for non-ideal features systematically observed in the voltammetric curves of **S-2** samples prepared on lowly-doped n-Si and a-Si samples. These features are ascribed to electrostatic interactions arising from the diode nature of the semiconductor electrode and surface charges from the molecular adsorbate. This model is based on the one developed by Santangelo *et al.*[13] in 1988 and expanded to take into account electrostatic interactions using the model developed by Laviron with the refinements proposed by Aleveque[5] in the limit of fast kinetics. The model explained here describes the semiconductor/monolayer/electrolyte as two polarizable interfaces in series, as depicted in Supplementary Figure 23.

When a potential E is applied, the overall potential drop across the semiconductor/monolayer/electrolyte interface (which corresponds to the applied potential) is distributed as a potential difference across the semiconductor space-charge layer E_d and the potential across the monolayer/electrolyte interface E_e

$$E = E_d + E_e \quad \text{(Supplementary Equation 32)}$$

Where the current flowing is the same through each interface

$$I = I_d = I_e \quad \text{(Supplementary Equation 33)}$$

Now we develop the expressions for the current at each interface by considering the potential waveform of a cyclic voltammetry experiment.

Fast kinetics limit

When interactions are present, the intensity-potential relationship for the monolayer/electrolyte interface can be studied by using an explicit equation (Supplementary Equation 22) for expressing the current as a function of potential.

The semiconductor is assumed to behave as a diode, and the current-potential expression is given by using the diode current-voltage expression, which in the case of a photoanode is

$$I_{\mathrm{d}} = I_{\mathrm{L}} + I_0\left[1-\exp\left(-\frac{nFE_{\mathrm{d}}}{RTD}\right)\right] \quad \text{(Supplementary Equation 34)}$$

and for a photocathode

$$I_{\mathrm{d}} = I_{\mathrm{L}} + I_0\left[\exp\left(\frac{nFE_d}{RTD}\right)-1\right] \quad \text{(Supplementary Equation 35)}$$

where I_{L} and I_0 are the photogenerated current and the reverse saturation diode current, and D is the diode ideality factor.

(Supplementary Equation 34) and (Supplementary Equation 35) can be rewritten respectively as

$$\frac{I_{\mathrm{d}}}{I_{\mathrm{L}}+I_0} = 1-\exp\left[-\frac{nF}{RTD}\left(E_{\mathrm{e}}-E_{OC}\right)\right] \quad \text{(Supplementary Equation 36)}$$

and

$$\frac{I_d}{-I_{\mathrm{L}}+I_0} = \exp\left[\frac{nF}{RTD}\left(E_{\mathrm{e}}-E_{OC}\right)\right] -1 \quad \text{(Supplementary Equation 37)}$$

where E_{OC} for the photoanode is the open circuit potential given by

$$E_{\mathrm{OC}} = \frac{DRT}{nF}\ln\left(\frac{I_0}{I_{\mathrm{L}}+I_0}\right) \quad \text{(Supplementary Equation 38)}$$

and for the photocathode

$$E_{\mathrm{OC}} = \frac{DRT}{nF}\ln\left(\frac{|I_{\mathrm{L}}|+I_0}{I_0}\right) \quad \text{(Supplementary Equation 39)}$$

By combining (Supplementary Equation 21) and (Supplementary Equation 38) the current-potential expression for the photoanode is obtained as

$$\frac{I_d}{I_p} = \frac{1}{\theta}\left\{1-\exp\left[-\frac{nF}{DRT}\left(E_d-E_{OC}\right)\right]\right\} \quad \text{(Supplementary Equation 40)}$$

with

$$\theta = \frac{I_p}{I_{\mathrm{L}}+I_0} \quad \text{(Supplementary Equation 41)}$$

And for the photocathode, by combining (Supplementary Equation 21) with (Supplementary Equation 39),

$$\frac{I_d}{I_p} = \frac{1}{\theta}\left\{\exp\left[\frac{nF}{DRT}\left(E_d-E_{OC}\right)\right]-1\right\} \quad \text{(Supplementary Equation 42)}$$

with

$$\theta = \frac{I_p}{|I_L| + I_0}$$ (Supplementary Equation 43)

In order to obtain the current-potential curves at the semiconductor/monolayer/electrolyte system it is necessary to determine the values of the two potential drops, E_e and E_d. This can be done by equating (Supplementary Equation 22) and (Supplementary Equation 34) or (Supplementary Equation 35) for a photoanode or a photocathode, respectively, and considering (Supplementary Equation 32). If we solve for E_e for a photoanode

$$\pm\frac{(4-2G)\xi}{(1+\xi)^2 - 2G\xi} = \frac{1}{\theta}\left\{1-\exp\left[-\frac{nF}{DRT}(E - E_e - E_{OC})\right]\right\}$$ (Supplementary Equation 44)

whereas for a photocathode

$$\pm\frac{(4-2G)\xi}{(1+\xi)^2 - 2G\xi} = \frac{1}{\theta}\left\{1-\exp\left[-\frac{nF}{DRT}(E - E_e - E_{OC})\right]\right\}$$ (Supplementary Equation 45)

(Supplementary Equation 44) and (Supplementary Equation 45) can be solved numerically (for example, by using bisection method). Once E_e is obtained, by inserting its value into (Supplementary Equation 22) the current is obtained. The only variables to consider are the interaction parameters *G* and *s* and the diode parameter *θ*. In the case of inverted potential peaks, the value of *θ* changes from the anodic (direct) segment to the cathodic (reverse) one.

Finite kinetics limit

The analysis of the semiconductor/electrolyte interface done in the previous section (fast kinetics limit) is still valid but we must modify the treatment corresponding to the monolayer/electrolyte interface. In this case, the current is given by (Supplementary Equation 24). Moreover, the current in terms of the variation of surface coverage of species R can be written as

$$\frac{I}{nQ_F} = -v\frac{df_R}{dE}$$ (Supplementary Equation 46)

(Supplementary Equation 24) and (Supplementary Equation 40) can be re-written in a dimensionless way as

$$\psi = \frac{I}{I_p / H} = -(4-2G)\frac{df_r}{d\eta} = \\ = (4-2G)\bar{k}_{red}\left\{f_r e^{\eta} e^{-(y-G)-f_r(G+s)} - (1-f_r)e^{-(y-s)+f_r(G-s)}\right\}$$ (Supplementary Equation 47)

where η, I_p and G are given by (Supplementary Equation 14), (Supplementary Equation 21) and (Supplementary Equation 18), respectively. Moreover,

$$\bar{k}_{red} = \bar{k}_{et}e^{-\alpha\eta}$$ (Supplementary Equation 48)

In order to solve (Supplementary Equation 47), we can work for the potential E_d at the interface semiconductor solution in terms of the current in (Supplementary Equation 34) (photoanode) and (Supplementary Equation 35) (photocathode),

$$E_d = \begin{cases} E_{OC} + \dfrac{DRT}{nF}\ln\left(1+\theta\psi\right) & \text{photocathode} \\ E_{OC} - \dfrac{DRT}{nF}\ln\left(1-\theta\psi\right) & \text{photoanode} \end{cases} \qquad \text{(Supplementary Equation 49)}$$

By taking into account that

$$E_e - E^{0\prime} = E - E^{0\prime} - E_d \qquad \text{(Supplementary Equation 50)}$$

it can be written that

$$e^{\eta} \begin{cases} = \dfrac{e^{\eta_{eff}}}{\left(1+\theta\psi\right)^{D}} = \dfrac{e^{\eta_{eff}}}{\left(1-\theta\left(4-2G\right)\dfrac{df_r}{d\eta}\right)^{D}} & \text{photocathode} \\ = e^{\eta_{eff}}\left(1-\theta\psi\right)^{D} = e^{\eta_{eff}}\left(1+\theta\left(4-2G\right)\dfrac{df_r}{d\eta}\right)^{D} & \text{photoanode} \end{cases} \qquad \text{(Supplementary Equation 51)}$$

where

$$\eta_{eff} = \frac{nF}{RT}\left(E - E^{0\prime} - E_{OC}\right) \qquad \text{(Supplementary Equation 52)}$$

By inserting (Supplementary Equation 51) into (Supplementary Equation 47) two differential algebraic equations are obtained,

$$-\frac{df_r}{d\eta} = \begin{cases} \bar{k}^0 \dfrac{e^{-\alpha\eta_{eff}}}{\left(1-\theta\left(4-2G\right)\dfrac{df_r}{d\eta}\right)^{-\alpha D}} \left\{ \dfrac{f_r e^{-(y-G)-f_r(G+s)} e^{\eta_{eff}}}{\left(1-\theta\left(4-2G\right)\dfrac{df_r}{d\eta}\right)^{D}} - \left(1-f_r\right) e^{-(y-s)+f_r(G-s)} \right\} \\ \text{photocathode} \\ \bar{k}^0 e^{-\alpha\eta_{eff}} \left(1+\theta\left(4-2G\right)\dfrac{df_r}{d\eta}\right)^{-\alpha D} \left(f_r e^{-(y-G)-f_r(G+s)} e^{\eta_{eff}} \left(1+\theta\left(4-2G\right)\dfrac{df_r}{d\eta}\right)^{D} - \right. \\ \left. - \left(1-f_r\right) e^{-(y-s)+f_r(G-s)} \right) \qquad \text{photoanode} \end{cases}$$

(Supplementary Equation 53)

In order to solve numerically (Supplementary Equation 53), the simplest procedure is to use an Euler implicit approach in which the derivative is changed by

$$\left|\frac{df_r}{d\eta}\right| \approx \left|\frac{\Delta f_r}{\Delta\eta}\right| = \left|\frac{f_{r,i} - f_{r,i-1}}{\Delta\eta}\right| \qquad \text{(Supplementary Equation 54)}$$

where subscript "i" indicates the potential being considered.

By inserting (Supplementary Equation 54) into (Supplementary Equation 53) an algebraic implicit equation is obtained that should be solved iteratively by using, for example, a bisection routine. Since for the initial potential of the scan the value of f_r is known (i.e., $f_{r,0} = 1$, with $i = 0$ indicating the initial potential of the scan), the only remaining task is to work for $f_{r,i}$ with $i \geq 1$. Supplementary Fig. 14 shows the influence of the rate constant on the cyclic voltammetry curves.

Supplementary Note 7

The transition from the N to the NR region is arbitrarily chosen and only intended to show where electrode kinetics starts to mask the electrostatic effect on the "peak potential inversion". The electrostatic effect on the current-potential curves is always present but it can often be masked by slow kinetics of charge-transfer.

Supplementary Note 8: Glossary

Term	Description	Definition
A	area of the electrode	
a_o	interaction parameter between oxidized species (o-o)	
a_{or}	interaction parameter for oxidized with reduced species (o-r)	
a_r	interaction parameter between reduced species (r-r)	
b_o	adsorption coefficient for the oxidized species	
b_r	adsorption coefficient for the reduced species	
D	diode ideality factor	
d_{ML}	monolayer thickness	
E	applied potential	
$E^{0'}$	formal potential	
E_d	potential drop across the space charge layer	
E_e	potential drop across the monolayer/electrolyte interface	
E_f	final applied potential	
E_{fb}	flat band potential	
E_i	initial scan potential	
E_{OC}	open circuit potential	
E_p	peak potential	
$E_{p,a}$	potential of the peak current on the anodic scan	
$E_{p,c}$	potential of the peak current on the cathodic scan	
F	Faraday constant (96500 F)	
f_o	fractional oxidized coverage	$f_o= \Gamma o/\Gamma_T$
f_r	fractional reduced coverage	$f_r= \Gamma_r/\Gamma_T$
fwhm	full width of half maximum peak current	

G	overall interaction parameter	$G = a_o + a_r - 2a_{or}$
H		$H = \frac{RT}{n^2Fv}\frac{1}{Q_F}$
I	current intensity	
I_0	reverse saturation current	
I_d	current flowing across the space charge layer	
I_e	current flowing across the monolayer/electrolyte interface	
I_L	photogenerated current	
I_p	peak current	
I_α	photoelectron counts for the element α	
J		$J = \exp\left(\frac{nF}{RT}\left(E(t) - E^{0'}\right)\right)$
j	number of water molecules displaced by one molecule of "o" or "r"	
k_{et}	Rate constant for electro-reduction or electro-oxidation processes at $E = E^{0'}$	
k_{red}	rate constant for electro-reduction processes (in s^{-1})	$k_{red} = k_{et}e^{-\alpha\eta}$
$\bar{k}_{et}$		$\bar{k}_{et} = \frac{k_{et}}{\left(\frac{nFv}{RT}\right)}$
$\bar{k}_{red}$	Dimensionless rate constant for electro-reduction processes	$\bar{k}_{red} = \bar{k}_{et}e^{-\alpha\eta}$
n	number of electron per mol transferred	
N_C	number of carbon atoms in the molecule	
N_{ML}/N_{Si}	fractional monolayer (ML) coverage	
N_{Si}	number density of atoms for the Si(100) surface	
Q_F	faradaic charge	$Q_F = FA\Gamma_T$
R	gas constant	
s		$s = a_r - a_o$
SFα	instrument-specific sensitivity factor for the element α	
T	temperature (298 K)	
t	time	
t_{inv}	time at which the scan is inverted	

v	scan rate	
y		$y = a_o + a_r$
α	coefficient of symmetry/charge-transfer	
Γ	total coverage	
Γ_o	coverage of the oxidized species	
Γ_r	coverage of the reduced species	
η		$\eta = \frac{nF}{RT}\left(E - E^{0'}\right)$
θ	takeoff angle between the surface and the analyzer	
θ		$\theta = \frac{I_p}{I_L + I_0}$
$\lambda_{\alpha,\beta}$	inelastic mean free path of electrons emitted from α travelling through β	
ξ		$\xi = \exp\left(\frac{nF}{RT}\left(E - E_p\right) + \frac{2G}{1 + exp\left(-\frac{nF\left(E - E_p\right)}{\left(1 - 0.4G\right)RT}\right)}\right)$
ρ_{Si}	Si atoms per unit of volume	
Φ_i	electric potential	
ψ	dimensionless current	

Supplementary Methods: XPS surface characterization. S-2 samples.

Analysis of the XPS spectra involved background subtraction using the Shirley routine and a subsequent nonlinear least-squares fitting to mix Gaussian−Lorentzian functions. A variable weighting of these functions was used depending on the specific envelope. Data from the C 1s emission region were fitted to functions having 70% Gaussian and 30% Lorentzian character. The N 1s spectral region was fitted to two peaks held 1.2 eV apart, each composed of a 100% Gaussian line shape. Signals from the Si 2p emission were fitted to two 95% Gaussian and 5% Lorentzian functions held 0.6 eV apart, 0.6 eV in fwhm's and with the Si $2p_{1/2}$:Si $2p_{3/2}$ peak area ratio kept at 1:2. The same line shape was used for SiO_x signals (Si^{+}–Si^{4+}) but constrains on fwhm's and splitting were removed. The Fe 2p spectral region was deconvoluted with functions having a 30% Gaussian and 70% Lorentzian line shape. Atomic compositions of the surface samples were corrected for the number of scans accumulated and for the atomic sensitivity of the element.

Narrow scans in the XPS spectra (Supplementary Fig. 25) of "as-prepared" surfaces obtained by reaction of azide **2** with acetylene-terminated monolayers (**S-1** samples) support a positive outcome for the formation of surface triazoles bearing ferrocene tethers (**S-2** samples). All of the spectral data match literature data for analogous films prepared on Si(100) electrodes.[14-16] High resolution XPS scan of the Fe 2p region in Supplementary Fig. 25a shows the two major spin-orbit split components Fe $2p_{3/2}$ and Fe $2p_{1/2}$ at 708.3 and 721.1 eV, respectively, thus indicating a predominant Fe(II) population with no clear evidence of Fe(III) species. Narrow N 1s scans (Supplementary Fig. 25b) show a broad signal centred at ca. 401 eV that best fits to two functions held 1.2 eV apart (400.4 and 401.6 eV, 2:1 ratio of the integrated areas)[17] and is associated with photoelectrons emitted from nitrogen atoms of the triazole cycle. Both the observed binding energies and fwhm's (ca. 1.6 eV for both functions)[18] compared well with that observed for other triazoles prepared surfaces.[19] The assignment of the N 1s signals is also supported by data and DFT calculations for aromatic compounds containing sp^2 N atoms bonded to two or three atoms, with the N 1s signal from the former being ca. 1 eV lower than the latter.[20,21] Absent from the N 1s spectra is the high binding energy signal (ca. 404 eV) corresponding to the electron-deficient nitrogen atom in the azido group, suggesting no physisorption of azide **2** in the samples.[22] Emissions from the C 1s core levels (Supplementary Fig. 25c) were deconvoluted and fitted to three functions: i) an aliphatic and aromatic carbon-bonded carbon (C−C)[23,24] peak centered at 285.0 eV (78% of total carbon, 1.2 eV fwhm); ii) a nitrogen- or oxygen-bonded carbon (C−N/−O)[18,25,26] peak at ca. 286.3 eV (15%, 1.7 eV fwhm); and iii) a lower binding energy component at 283.6 eV (7%, 1.1 eV fwhm) assigned to a carbons from a silylated olefin (Si–C̲=C, Si–C in figure)[27,28]. The observed binding energies were in good agreement with those reported for the corresponding carbons in other immobilized triazoles.[14,18] Spectroscopic shifts for the Si 2p region introduced by Si(1)-Si(4) oxides are extensively documented in literature.[29,30] Typical XPS Si 2p narrow scans for the as-prepared ferrocene-modified surface, as in Supplementary Fig. 25d as well as in Fig. 3d of the main text, lack of chemically shifted emissions associated with silicon oxide species (102-104 eV). The absence of oxides indicates a high quality of the functionalized surface (**S-2**).[31]

Further, quantitative considerations of the C 1s and N 1s regions support the formation of a **S-2** with representative elemental ratios, such as the Fe:N, showing experimental values in very good agreement with those predicted by the stoichiometry of atoms on the surface (experimental 1.0:3.4 vs. stoichiometric 1.0:3.0). This ratio decreases upon the anodic treatment of the samples (Supplementary Fig. 11) suggesting losses of Fc units – reinforcing on similar conclusions drawn for the electrochemical measurements (i.e. drop in ferrocene coverages by

measurements of charges exchanged by the electrode). An increase in the Si:C ratio upon the anodic experiments (1.8, "as prepared"; 2.0, "narrow waves"; and 2.0 for the "over-oxidized" electrodes) indicates that the damages to the Fc units are possibly paralleled by damages or loss to the whole carbonaceous film. Further experiments will be needed to clarify on the molecular details of the monolayer loss.

Changes in the reaction time for the visible light assisted hydrosilylation of 1,8-nonadiyne on Si(111).

As discussed in the experimental section (main text), the visible light-assisted hydrosilylation of 1,8-nonadiyne to prepare **S-1** samples on Si-H was routinely stopped after a 2 h reaction period. A representative set of XPS spectra for these samples is found in Supplementary Fig. 12 (data in the left column) as well as in Fig. 3 of the main text. The high-resolution C 1s signal was generally fitted with three components; a major contribution at 285.0 eV (74%, 1.3 eV fwhm) assigned to the methylene carbons of the adsorbate,[26] a low binding energy peak at 283.7 eV (12%, 1.0 eV fwhm) assigned to a carbons from a silylated olefin (Si–C=C,[27,28] on hydrogenated silicon surfaces, the assembly of monolayers of 1-alkynes is likely to result in sp^2 hybridization at the surface[32]) and high binding energy component at 286.1 eV (14%, 1.3 eV fwhm) that can tentatively be assigned to either an asymmetry in the main peak,[33] oxygen-bound carbon atoms (C–O),[28] or sp-hybridized carbon-bound carbons.[34] Absent from the spectra are Si 2p emissions associated with silicon oxide species (102-104 eV). With regards to adventitious C–O bonds, we note that although reported in several works, the precise chemical nature of the C–O bonds is still being debated.[26,28,33,35] Kramer and co-workers suggests that a shift to ca. 286 eV is most likely associated to monohydroxyl carbon atoms (–Si–CHC(OH)–).[33] More recently Zuilhof and co-workers have advanced the hypothesis that this high BE emission may also come from –C≡C– groups.[34]

Along with the spectral data for **S-1** samples prepared by shorter hydrosilylation reaction times (2 min and 10 min) in Supplementary Fig. 12 we also present the estimated values of fractional monolayer (ML) coverage (N_{ML}/N_{Si}) obtained from XPS data according to the model of Cicero *et al.*[2]:

$$\frac{N_{ML}}{N_{Si}} = \frac{\lambda_{Si,Si}\, d_{ML}}{\lambda_{C,ML}\, N_C} \frac{I_C / SF_C}{I_{Si} / SF_{Si}} \frac{\rho_{Si}}{N_{Si}} \frac{\exp[-d_{ML} / (\lambda_{Si,ML} \sin\theta)]}{1-\exp[d_{ML} / (\lambda_{C,ML} \sin\theta)]}$$

(Supplementary Equation 55)

where d_{ML} is the monolayer thickness obtained from XRR,[18] N_C is the number of carbon atoms in the molecule, $\lambda_{\alpha,\beta}$ is the inelastic mean free path of electrons emitted from α travelling through β ($\lambda_{Si,Si}$ = 16 Å, $\lambda_{C,ML}$ = 36 Å, $\lambda_{Si,ML}$ = 41.5 Å),[2] I_α and SF_α are, respectively, the photoelectron counts and the instrument-specific sensitivity factor for the element α, ρ_{Si} is the Si atoms per unit of volume (0.05 $Å^{-3}$), N_{Si} is the number density of atoms for the Si(111) surface (0.078 $Å^{-2}$), θ is the takeoff angle between the surface and the analyzer (90° in our case).

Supplementary References

1 Fabre, B. Functionalization of oxide-free silicon surfaces with redox-active assemblies. *Chem. Rev.* **116**, 4808–4849 (2016).

2 Cicero, R. L., Linford, M. R. & Chidsey, C. E. D. Photoreactivity of unsaturated compounds with hydrogen-terminated silicon(111). *Langmuir* **16**, 5688–5695 (2000).

3 Laviron, E. General expression of the linear potential sweep voltammogram in the case of diffusionless electrochemical systems. *J. Electroanal. Chem. Interf. Electrochem.* **101**, 19–28 (1979).

4 Laviron, E. Surface linear potential sweep voltammetry: equation of the peaks for a reversible reaction when interactions between the adsorbed molecules are taken into account. *J. Electroanal. Chem. Interf. Electrochem.* **52**, 395–402 (1974).

5 Alévêque, O. & Levillain, E. A generalized lateral interactions function to fit voltammetric peaks of self-assembled monolayers. *Electrochem. Commun.* **67**, 73–79 (2016).

6 Laviron, E. & Roullier, L. General expression of the linear potential sweep voltammogram for a surface redox reaction with interactions between the adsorbed molecules: applications to modified electrodes. *J. Electroanal. Chem. Interf. Electrochem.* **115**, 65–74 (1980).

7 Laborda, E., Henstridge, M. C., Batchelor-McAuley, C. & Compton, R. G. Asymmetric Marcus-Hush theory for voltammetry. *Chem. Soc. Rev.* **42**, 4894–4905 (2013).

8 Nelson, A. Co-refinement of multiple-contrast neutron/X-ray reflectivity data using MOTOFIT. *J. Appl. Crystallogr.* **39**, 273–276 (2006).

9 Névot, L. & Croce, P. Caractérisation des surfaces par réflexion rasante de rayons X. Application à l'étude du polissage de quelques verres silicates. *Rev. Phys. Appl. (Paris)* **15**, 761–779 (1980).

10 Cowley, R. A. & Ryan, T. W. X-ray scattering studies of thin films and surfaces: thermal oxides on silicon. *Journal of Physics D: Applied Physics* **20**, 61 (1987).

11 Roman, A. J., Sevilla, J. M., Pineda, T. & Blázquez, M. A study on maxima and inverted peaks in cyclic voltammetry. Electrochemical reduction of pyridine-4-aldoxime at an HMDE. *Journal of Electroanalytical Chemistry* **517**, 15–19 (2001).

12 Miah, M. R. & Ohsaka, T. Observation of 'inverted peak' during molecular oxygen reduction at Au electrode in alkaline media. *Electrochimica Acta* **52**, 6378–6385 (2007).

13 Santangelo, P. G., Miskelly, G. M. & Lewis, N. S. Cyclic voltammetry at semiconductor photoelectrodes. 1. ideal surface-attached redox couples with ideal semiconductor behavior. *J. Phys. Chem.* **92**, 6359–6367 (1988).

14 Ciampi, S., Le Saux, G., Harper, J. B. & Gooding, J. J. Optimization of click chemistry of ferrocene derivatives on acetylene-functionalized silicon(100) surfaces. *Electroanal.* **20**, 1513–1519 (2008).

15 Ciampi, S. et al. Silicon (100) electrodes resistant to oxidation in aqueous solutions: an unexpected benefit of surface acetylene moieties. *Langmuir* **25**, 2530–2539 (2009).

16 Ciampi, S., James, M., Michaels, P. & Gooding, J. J. Tandem "Click" reactions at acetylene-terminated Si(100) monolayers. *Langmuir* **27**, 6940–6949 (2011).

17 Li, Y., Wang, J. & Cai, C. Rapid grafting of azido-labeled oligo(ethylene glycol)s onto an alkynyl-terminated monolayer on nonoxidized silicon via microwave-assisted "Click" reaction. *Langmuir* **27**, 2437–2445 (2011).

18 Ciampi, S. et al. Functionalization of acetylene-terminated monolayers on Si(100) surfaces: a click chemistry approach. *Langmuir* **23**, 9320–9329 (2007).

19 Li, Y., Zhao, M. R., Wang, J., Liu, K. & Cai, C. Z. Biofunctionalization of a "Clickable" organic layer photochemically grafted on titanium substrates. *Langmuir* **27**, 4848–4856 (2011).

20 Alfredsson, Y. et al. Electronic structure of a vapor-deposited metal-free phthalocyanine thin film. *J. Chem. Phys.* **122**, 214723–214728 (2005).

21 Ito, E. et al. Soft X-ray absorption and X-ray photoelectron spectroscopic study of tautomerism in intramolecular hydrogen bonds of N-salicylideneaniline derivatives. *J. Am. Chem. Soc.* **119**, 6336–6344 (1997).

22 Devadoss, A. & Chidsey, C. E. D. Azide-modified graphitic surfaces for covalent attachment of alkyne-terminated molecules by "Click" chemistry. *J. Am. Chem. Soc.* **129**, 5370–5371 (2007).

23 Lehner, A., Steinhoff, G., Brandt, M. S., Eickhoff, M. & Stutzmann, M. Hydrosilylation of crystalline silicon (111) and hydrogenated amorphous silicon surfaces: a comparative X-ray photoelectron spectroscopy study. *J. Appl. Phys.* **94**, 2289–2294 (2003).

24 Cerofolini, G. F. et al. Hydrosilation of 1-alkyne at nearly flat, terraced, homogeneously hydrogen-terminated silicon (100) surfaces. *Surf. Interface Anal.* **36**, 71–76 (2004).

25 Böcking, T., James, M., Coster, H. G. L., Chilcott, T. C. & Barrow, K. D. Structural characterization of organic multilayers on silicon(111) formed by immobilization of molecular films on functionalized Si-C linked monolayers. *Langmuir* **20**, 9227–9235 (2004).

26 Wallart, X., de Villeneuve, C. H. & Allongue, P. Truly quantitative XPS characterization of organic monolayers on Silicon: study of alkyl and alkoxy monolayers on H-Si(111). *J. Am. Chem. Soc.* **127**, 7871–7878 (2005).

27 Yaffe, O. et al. Hg/molecular monolayer − Si junctions: electrical interplay between monolayer properties and semiconductor doping density. *J. Phys. Chem. C* **114**, 10270–10279 (2010).

28 Scheres, L., Arafat, A. & Zuilhof, H. Self-assembly of high-quality covalently bound organic monolayers onto silicon. *Langmuir* **23**, 8343–8346 (2007).

29 Himpsel, F. J., McFeely, F. R., Taleb-Ibrahimi, A., Yarmoff, J. A. & Hollinger, G. Microscopic structure of the SiO_2/Si interface. *Phys. Rev. B* **38**, 6084–6096 (1988).

30 Cerofolini, G. F., Galati, C., Reina, S. & Renna, L. Grafting of 1-alkynes to hydrogen-terminated (100)silicon surfaces. *Appl. Phys. A - Mater.* **80**, 161–166 (2005).

31 Sieval, A. B., Linke, R., Zuilhof, H. & Sudhölter, E. J. R. High-quality alkyl monolayers on silicon surfaces. *Adv. Mater.* **12**, 1457–1460 (2000).

32 Walsh, M. A., Walter, S. R., Bevan, K. H., Geiger, F. M. & Hersam, M. C. Phenylacetylene one-dimensional nanostructures on the Si(100)-2 × 1:H surface. *J. Am. Chem. Soc.* **132**, 3013–3019 (2010).

33 Kondo, M., Mates, T. E., Fischer, D. A., Wudl, F. & Kramer, E. J. Bonding structure of phenylacetylene on hydrogen-terminated Si(111) and Si(100): surface photoelectron spectroscopy analysis and ab initio calculations. *Langmuir* **26**, 17000–17012 (2010).

34 Rijksen, B. et al. Hexadecadienyl monolayers on hydrogen-terminated Si(111): faster monolayer formation and improved surface coverage using the enyne moiety. *Langmuir* **28**, 6577–6588 (2012).

35 Cerofolini, G. F., Galati, C., Reina, S. & Renna, L. Quantitative XPS analysis of hydrosilated 1-alkene and 1-alkyne at terraced, dihydrogen-terminated, 1 × 1 (100) silicon. *Surf. Interface Anal.* **38**, 126–138 (2006).
